# Agricultural Landscape Heterogeneity Matter: Responses of Neutral Genetic Diversity and Adaptive Traits in a Neotropical Savanna Tree

**DOI:** 10.3389/fgene.2020.606222

**Published:** 2021-02-04

**Authors:** Tatiana Souza do Amaral, Juliana Silveira dos Santos, Fernanda Fraga Rosa, Marcelo Bruno Pessôa, Lázaro José Chaves, Milton Cezar Ribeiro, Rosane Garcia Collevatti

**Affiliations:** ^1^Laboratório de Genética & Biodiversidade, ICB, Universidade Federal de Goiás (UFG), Goiânia, Brazil; ^2^Laboratório de Ecologia Espacial e Conservação (LEEC), Departamento de Biodiversidade, Universidade Estadual Paulista (UNESP), Rio Claro, Brazil; ^3^Laboratório de Metacomunidades e Paisagem, ICB, Universidade Federal de Goiás (UFG), Goiânia, Brazil; ^4^Escola de Agronomia, Universidade Federal de Goiás (UFG), Goiânia, Brazil

**Keywords:** agroecosystem, Caryocaraceae, Cerrado, fragmentation, genetic diversity, landscape genetics, model selection, quantitative genetics

## Abstract

Plants are one of the most vulnerable groups to fragmentation and habitat loss, that may affect community richness, abundance, functional traits, and genetic diversity. Here, we address the effects of landscape features on adaptive quantitative traits and evolutionary potential, and on neutral genetic diversity in populations of the Neotropical savanna tree *Caryocar brasiliense*. We sampled adults and juveniles in 10 savanna remnants within five landscapes. To obtain neutral genetic variation, we genotyped all individuals from each site using nine microsatellite loci. For adaptive traits we measured seed size and mass and grown seeds in nursery in completely randomized experimental design. We obtained mean, additive genetic variance (*V*_*a*_) and coefficient of variation (*CV*_*a*_%), which measures evolvability, for 17 traits in seedlings. We found that landscapes with higher compositional heterogeneity (SHDI) had lower evolutionary potential (*CV*_*a*_%) in leaf length (LL) and lower aboveground dry mass (ADM) genetic differentiation (*Q*_*ST*_). We also found that landscapes with higher SHDI had higher genetic diversity (*He*) and allelic richness (*AR*) in adults, and lower genetic differentiation (*F*_*ST*_). In juveniles, SHDI was also positively related to *AR*. These results are most likely due to longer dispersal distance of pollen in landscapes with lower density of flowering individuals. Agricultural landscapes with low quality mosaic may be more stressful for plant species, due to the lower habitat cover (%), higher cover of monocropping (%) and other land covers, and edge effects. However, in landscapes with higher SHDI with high quality mosaic, forest nearby savanna habitat and the other environments may facilitate the movement or provide additional habitat and resources for seed disperses and pollinators, increasing gene flow and genetic diversity. Finally, despite the very recent agriculture expansion in Central Brazil, we found no time lag in response to habitat loss, because both adults and juveniles were affected by landscape changes.

## Introduction

Impacts of fragmentation and habitat loss on plant genetic diversity are still poorly understood, despite the increase in the number of studies in recent years (Storfer et al., 2010; Manel and Holderegger, 2013). Several works point out the decreasing in genetic diversity due to fragmentation process (e.g., Jump and Peñuelas, 2006; Aparicio et al., 2012; Carvalho et al., 2015; Gómez-Fernández et al., 2016; Collevatti et al., 2020a), while others found no reduction in genetic diversity (e.g., Hall et al., 1996; Collevatti et al., 2001; Bacles et al., 2005; Winkler et al., 2011; Carvalho et al., 2019; Soares et al., 2019). The variation in fragmentation and habitat loss effects on genetic diversity may be due to differences in life history because each species respond to landscape changes according to their dispersal capacity and ecological requirements (Prevedello and Vieira, 2010; Eycott et al., 2012).

In plants, landscape structure may affect connectivity due to the influence on germination and establishment (Soons and Heil, 2002; Auffret et al., 2017). Additionally, plants depend on animals for pollen and seed dispersal, thus the response to landscape changes will also relies on how pollinators and seed disperses perceive environmental modifications (García et al., 2007; Carvalho et al., 2015; Auffret et al., 2017; Uroy et al., 2019), which may hinder the detection of habitat loss and fragmentation effects on plant genetic diversity. Moreover, plants may have long life cycle, specially trees, and time since fragmentation may not have been sufficient to cause a decrease in genetic diversity (Collevatti et al., 2001; Kramer et al., 2008). Thus, genetic diversity and differentiation in adult trees may be the outcome of past environmental changes and not recent or ongoing changes in landscape (Collevatti et al., 2001; Kramer et al., 2008). In fact, some studies have shown significant effects of fragmentation process, decreasing genetic diversity in seedlings, but not in adult trees (Sebbenn et al., 2011; Quesada et al., 2013; Martins et al., 2016), suggesting a time lag for ongoing habitat fragmentation be detected in adults (e.g., Aguilar et al., 2008). Therefore, comparing the effects of landscape changes in genetic diversity of seedlings and adults may give clues on the different roles of ongoing habitat loss and fragmentation and past demographic history.

The effects of fragmentation and habitat loss on plant genetic diversity are usually addressed using genetic variation at neutral loci, such as highly polymorphic microsatellites (e.g., Schmidt et al., 2009; Carvalho et al., 2015; Soares et al., 2019). Because of the high mutation rates, microsatellites usually display high levels of within-population heterozygosity (Hedrick, 1999), thus the effect of habitat loss on genetic diversity may only be detected after a certain threshold of population size is attained (Collevatti et al., 2001). On the other hand, quantitative adaptive traits evolve under genetic drift and selection (McKay and Latta, 2002) and though may respond faster to landscape changes. The loss of genetic variability at adaptive loci may lead to the loss of individual fitness and in population evolutionary potential (Lande, 1988; Reed and Frankham, 2001; Bijlsma and Loeschcke, 2012), being more informative to the understanding of the effects of land use in population long-term persistance (Holderegger et al., 2006, 2010). Therefore, assessing adaptive quantitative genetic variation and neutral genetic diversity are of utmost importance to the understanding of the effects of landscape changes on populations and to drive conservation strategies (Carvajal-Rodríguez et al., 2005).

In the last 50 years the Cerrado biome has been intensively cleared, losing almost half of its original area (Sano et al., 2010; Alencar et al., 2020). The Brazilian Cerrado biome is the largest Neotropical savanna and one of the world’s biodiversity hotspots because of its high level of endemism and threatening (Myers et al., 2000). It is one of the most important Brazilian agribusiness regions, and because of that only 7.5% of its area is legally conserved within public protected areas (Strassburg et al., 2017; Rosa, 2020). Currently, only 20% of farmland area within Cerrado biome must be protected in Legal Reserves (Brasil, 2012), which threats remnants of ecosystems and biodiversity outside protected areas (Metzger, 2010; Vieira et al., 2018). With the ongoing threats to Brazilian biodiversity, including the new perspective of legal reserves cut down (see Metzger et al., 2019), the understanding of how landscape changes and agroecosystems affect biodiversity is critical to develop better and cost-effective management and conservation strategies (Santos et al., 2020a).

**FIGURE 1:**
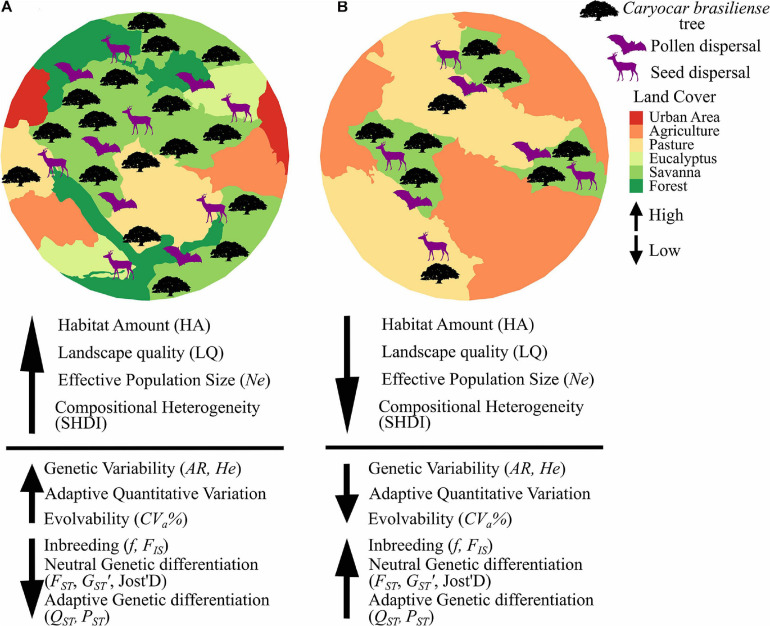
Predictions of expected effects of landscape composition and configuration and effective population size on neutral and adaptive quantitative genetic variables in *Caryocar brasiliense.* Because landscape may affect pollen and seed dispersal and germination and establishment of *C. brasiliense*, we hypothesize that **(A)** landscapes with higher habitat cover (HA), compositional heterogeneity (SHDI), landscape quality (LQ) and effective population size (*Ne*) have higher neutral (*AR*, *He*) and quantitative genetic variability and evolvability (*CV*_*a*_%), and lower genetic differentiation (*F_*ST*_, G_*ST*_’, Jost’D, Q_*ST*_, P_*ST*_*) and inbreeding (*f, F_*IS*_*). On the other hand, **(B)** landscapes with lower HA, SHDI, LQ, *Ne* have lower neutral and quantitative genetic variability and evolvability, and higher genetic differentiation and inbreeding.

Here we address the effects of landscape changes on a tree species, *Caryocar brasiliense* Cambess (Caryocaraceae), endemic to the savannas of Cerrado biome. It has hermaphroditic flowers, mixed-mating reproductive system (Collevatti et al., 2001, 2010a) and is pollinated by bats, mainly *Glossophaga soricina* and *Anoura geoffroyi* (Gribel and Hay, 1993). Seeds are dispersed mainly by mammals and large birds, such as deer (*Mazama americana* and *Mazama gouazoupira*), cotia (*Dasyprocta* spp.), tapir (*Tapirus terrestris*), maned wolf (*Chrysocyon brachyurus*), the greater rhea (*Rhea americana*), and jackdaw (*Cyanocorax cristatellus*) (Collevatti et al., 2010b; Zardo and Henriques, 2011). We specifically analyzed how landscape composition and configuration affects variation at adaptive quantitative traits and at neutral microsatellite markers using a multi-scale approach. Because landscape structure may affect seed dispersal distance, seed germination and establishment we hypothesize that populations in landscapes with higher habitat cover (%), compositional heterogeneity, landscape quality and connectivity have (i) higher genetic variability at neutral and (ii) adaptive quantitative loci and (iii) lower genetic differentiation and (iv) inbreeding (see our predictions in Figure 1). We also hypothesize that (v) population evolvability will be reduced due to habitat and connectivity losses following the trends expected for genetic variability. To account for the effects of genetic drift (Wright, 1931; Kimura, 1983), we analyze the effect of effective population size in adaptive and neutral genetic variability. Finally, because of time-lag effect on genetic diversity, we analyze the effects of landscape changes on genetic diversity at neutral loci at both adult trees and juveniles. Because of the recent history of Cerrado fragmentation (<60 years), we expect (vi) to find significant reduction in genetic diversity at neutral loci in juveniles but not in adults.

## Materials and Methods

### Study Sites and Landscape Metrics

**FIGURE 2:**
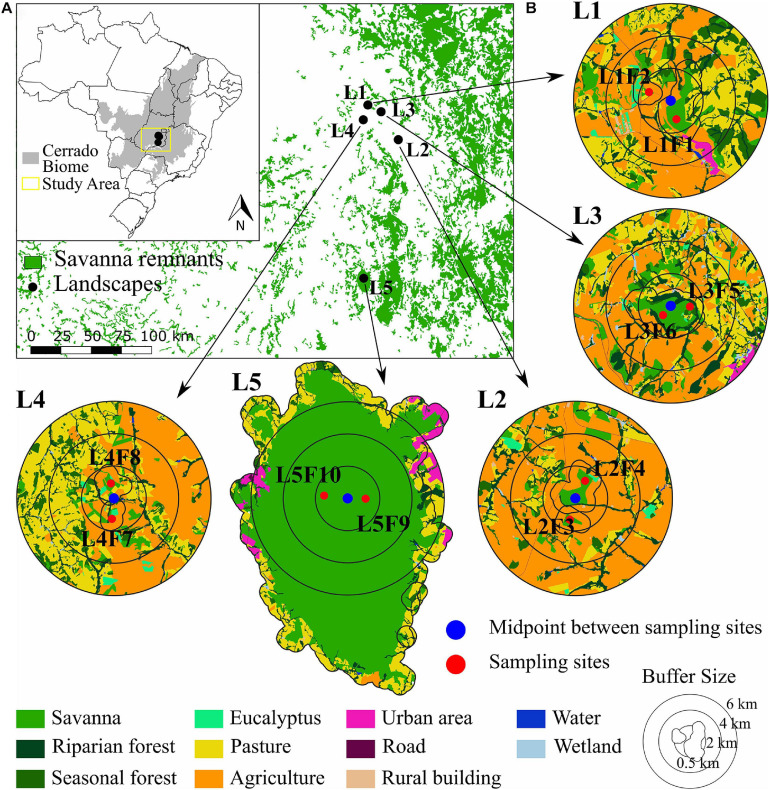
Geographical distribution of the five landscapes and 10 sampling sites of *Caryocar brasiliense* in the Cerrado biome. **(A)** The distribution of the Cerrado biome in Brazil (gray) and the five landscapes. **(B)** The five landscapes and sampling sites (red dots). The multi-scale landscapes are represented by buffers of 2, 4, and 6 km created around the midpoint (blue triangle) between the two sites within each landscape. Inner polygons around sampling sites are the 0.5 km buffers for node analyses. Land use categories are in legends.

We sampled 10 sites within five landscapes in the Cerrado biome, Goiás State, Brazil (Figure 2). The study is part of a Long-term Ecological Research (LTER) the COFA project (Functional connectivity in agricultural landscapes) implemented in a Brazilian intensive farming landscape. The region is dominated mainly by soybean, corn and pasture (Alencar et al., 2020). Soybean is mostly associated with corn in succession crops, which makes the corn spatial distribution the same as that of the soybean. Each landscape was defined by a circle of 2 km radius around a midpoint between two samplings sites with populations of *Caryocar brasiliense* (Figure 2B), with 1.4 km minimum distance between sampling sites. The 2 km landscapes had a gradient in the savanna cover ranging from 15 to 100% (Supplementary Table 1). Six sampling sites were structurally distinct patches of savanna (landscapes L1, L2, and L4, Figure 2B) and two pairs of sampling sites were within the same patches (landscapes L3 and L5, Figure 2B) in a large savanna protected area (Supplementary Table 1).

In each landscape, we mapped land cover using high resolution imagery available at the database of the Geographic Information System in ArcGis v.9.3 environment (Esri^®^). Mapping and classification were performed manually at 1:5,000 scale in the screen, followed by extensive field checking and validation. We mapped 11 land cover classes: (i) water courses; (ii) savanna; (iii) riparian forest; (iv) seasonal forest; (v) wetland; (vi) pasture; (vii) agriculture; (viii) rural building; (ix) urban area; (x) roads and train rails; and (xi) *Eucalyptus* spp. plantation (Figure 2B). In these landscapes, the agriculture land cover encompasses intensive soybean cultivation in the spring and summer (from October to March, the wet season), and the cultivation of corn in the summer and autumn (from February to June, the wet and end of the wet season), with a short period of fallow in the winter, from June to September (dry season). The pasture comprehends intensive managed grass for free-grazing livestock, mainly milk, and dairy cattle. However, pastures are mainly in high-slope landscapes and tend to have higher natural vegetation amount than landscapes dominated by agriculture (see Santos et al., 2020a).

We quantified landscape variables at node and link levels (Wagner and Fortin, 2013). To calculate landscape metrics at the node level, we drew buffers of 0.5 km around each focal site (Figure 2B). We chose this distance based on pollen dispersal distance (at least 500 m, Collevatti et al., 2010a) and genetic neighborhood size (∼ 86 m). Sites in protected areas (landscapes L3 and L5, Figure 2B) had the same landscape metrics. At the link level, we identified the midpoint between the two sampling sites and performed multi-scale analysis with buffers of 2, 4, and 6 km around each midpoint (Figure 2B). We chose these buffers because of the pollen dispersal distance and to avoid overlap among landscapes, therefore minimizing spatial auto-correlation effects on our analysis (Fortin and Dale, 2005).

To quantify habitat cover (%), we calculated the percentage of savanna at both node (Supplementary Table 2) and link levels (Supplementary Table 3). We also calculated functional connectivity, which focus on the landscape connectivity from the perspective of the species due to dispersal ability (Nathan et al., 2008). At node level, we summed the area (in hectares) of the patches of savanna with the focal site and the area of savanna patches connected by the 0.5 km buffers (Supplementary Table 2), following Collevatti et al. (2020a). At link level we summed the area (in hectares) of savanna patches in the 2, 4, and 6 km buffers (Supplementary Table 3). Functional connectivity was calculated using GRASS GIS 7.5 (GRASS Development Team, 2018).

Compositional heterogeneity was calculated using Shannon index (SHDI), including all land cover categories for both node and link levels (Supplementary Tables 2, 3). Analysis was performed with Fragstats software (McGarigal et al., 2012). Landscape quality (LQ) was calculated using the approach of Split Matrix Quality (SMQ) used on amphibians (Lion et al., 2014), similar to the influence of Matrix Permeability on primates (Silva et al., 2015). The LQ was calculated based on the percentage of each land cover class in each landscape (P_i_, Supplementary Tables 4, 5), and the quality score of each land cover class (Q_i_). To calculate quality scores, we considered the requirements and resources that each land cover can provide to both our focal tree species (*C. brasiliense*) and interacting species (i.e., pollinators and seed dispersers). Although *C. brasiliense* establishes in savanna (habitat), pollen (mainly bats) and seed dispersers (mammals and large frugivore birds) can perceive the landscape in different ways, using the anthropogenic matrix and forests as resource or as complementary habitat (Antongiovanni and Metzger, 2005; Anderson et al., 2007; Lyra-Jorge et al., 2008; Prevedello and Vieira, 2010). Thus, the delimitation of habitat may vary among interacting species and may be affected by the amount of different land covers (Metzger, 2001). We obtained Q_i_ for the main pollinator (*Glossophaga soricina*) and large-sized seed dispersers (*Mazama americana, Mazama guazoupira*, *Dasyprocta* sp., *Tapirus terrestris*, and *Chrysocyon brachyurus*) based on expert opinion (Supplementary Tables 4, 5). LQ was calculated for each pollinator and seed disperser at both node and link levels, for the different spatial scales (Supplementary Tables 6, 7). As spatial scale we considered the buffer size radius (km), given a centroid of interest (i.e., site location). Then we performed a principal component analysis (PCA) with landscape quality score (Qi) for pollinator and seed dispersers for each spatial scale, to account for the interaction of pollinator and seed dispersers. We ranked the value of the first principal component that explained 54.4% of the variation, and rescaled it from 1 to 10 to obtain the resistance weight of each land use type (Supplementary Table 5). We then calculated the LQ using the PCA resistance value (Supplementary Tables 6, 7). LQ and PCA analyses were performed in R version 3.6.1 (R Core Team, 2019).

### Adaptive Quantitative Trait Variation

We measured adaptive quantitative traits related to fitness of seeds and seedlings. Seed size represents the amount of resources that mother-tree invests in offspring, affecting germination success and seedling fitness (Leishman et al., 2000). Besides this, savanna species have developed several strategies to tolerate water deficit during dry seasons, low nutrient availability, high temperatures and frequent fires in the early stages of development (Hoffmann, 2000; Oliveira and Marquis, 2002; Hoffmann et al., 2004; Scariot et al., 2005). Savanna seedlings have a high investment in root growth to reach water table and root system also acts as a reserve organ allowing plant regrowth after disturbances such as fire (Hoffmann, 2000; Hoffmann and Franco, 2003; Hoffmann et al., 2004). This limits biomass accumulation in the shoot, which causes Cerrado species root/shoot ratio to be up 82% higher than forest species (Hoffmann and Franco, 2003). Savanna plants also invest more in stem diameter because thick bark provides protection from high temperatures during fire events (Hoffmann et al., 2003, 2009). Leaf characteristics can also influence plant responses to water deficit, as well as being responsible for light absorption and photosynthesis rates.

For seed sampling, we randomly chose 10 mother-trees in each sampling site (Supplementary Table 8), except for L1F2 (*n* = 8) and L2F6 (*n* = 2), due to the number of trees siring fruits. We obtained a total of 1,570 fruits (Supplementary Table 8), from which we obtained 2,561 ripened seeds. The number of seedlings analyzed per tree per site differed due to variation in germination (Supplementary Table 8). Each seed was measured and weighted to obtain seed traits (Supplementary Table 9): SLD (seed longitudinal diameter, mm), STD (seed transversal diameter, mm) and SM (seed mass, g). Seeds were grown in a greenhouse in a completely randomized experimental design and monitored daily to obtain the number of days to shoot (time to seed germination, TG) and the proportion of seeds that germinated (PG) (Supplementary Table 9).

After seed germination, we measured seedling aboveground height and stem diameter 76, 116, 133, and 145 days. We obtained height (HGR, cm/day) and diameter (DGR, mm/day) growth rates from the regression coefficient (b) and recorded the initial (76 days) and final (145 days) seedling height (IH and FH, cm) and diameter (ID and FD, mm). The number of leaves (NL), leaf length (LL) and width (LW) of each seedling was measured at the end of the experiment, 145 days after germination (Supplementary Table 9), from the mean value among three leaves per seedling. Seedlings were taken from the nursery pots after 145 days, to measure (Supplementary Table 9) aboveground shoot length (ASL, cm), root length (RL, cm), aboveground green mass (AGM, g) and dry mass (ADM, g), and root green mass (RGM, g) and dry mass (RDM, g).

To minimize correlation among quantitative variables, we performed Pearson correlation (r) analyses at both node (Supplementary Table 10) and link levels (Supplementary Table 11) and removed from subsequent analysis all variables we considered redundant (*r* > 0.5). After these steps, we kept for seed traits STD, and SM, and for seedlings, TG, LL, RDM, and ADM.

We estimated the mean and additive genetic variance (*Va*), and the narrow-sense heritability (*h*^2^) of each selected trait in each site. To address population evolutionary potential or evolvability we estimated the additive genetic coefficient of variation, *CV*_*a*_% (Houle, 1992; Hansen et al., 2011). To estimate these parameters at the node level, we used components of variance estimated by restricted maximum likelihood (REML) analysis, implemented at the model 82 in the software SELEGEN-REML/BLUP (Resende, 2016), for open-pollinated sib families with mixed-mating system. The estimates of additive variance and their derived parameters (*CV*_*a*_% and h^2^) were obtained by $\sigma_{A}^{2}=\frac{1}{2\,\theta }\,\sigma_{p}^{2}$, where $\sigma_{p}^{2}$ is the genetic variance among families and θ is the coancestry coefficient within family. We corrected the additive variance among sib families of mixed mating system populations assuming Wright’s equilibrium, using c = 2θ = $\frac{{\left(1+s\right)}^{2}}{2\,\left(2-s\right)}$, where *s* is the selfing rate (Vencovsky et al., 2001; Tambarussi et al., 2018). Under Wright’s equilibrium, $s=\frac{2\,f}{1+f}$ and $\dot{\theta }=\frac{{\left(1+3\,f\right)}^{2}}{8\,\left(1+f\right)}$ (Vencovsky and Crossa, 2003). The inbreeding coefficient (*f*) within population was obtained from juvenile’s microsatellite genotypes (see below). These parameters were estimated only for seedling traits obtained from controlled experiment in nursery, because seed traits were measured in seeds sampled in the field.

Quantitative genetic differentiation between pairs of sampling sites nested within landscape was estimated with *Q*_*ST*_ (Prout and Barker, 1993; Spitze, 1993) and *P*_*ST*_ (Leinonen et al., 2006). *Q*_*ST*_ is an estimator of the genetic differentiation of quantitative traits based on population additive genetic variance (Spitze, 1993) and was estimated for seedling traits measured in nursery under experimental conditions (TG, LL, RDM, and ADM). *P*_*ST*_ is analogous to *Q*_*ST*_ (Leinonen et al., 2006), but is used to quantify genetic differentiation among populations under uncontrolled environmental conditions when additive genetic component cannot be estimated. Therefore, we estimated *P*_*ST*_ for seed traits (SM and STD). *Q*_*ST*_ and *P*_*ST*_ parameters were calculated using variance components estimated using model 05 in the software SELEGEN – REML/BLUP (Resende, 2016), applying the correction for additive genetic variance for mixed mating system (see above).

### Neutral Genetic Variation

We sampled expanded leaves of adults (reproductive individuals) and juveniles of *C. brasiliense* in each sampling site for genetic analysis of neutral loci (Supplementary Table 8). Genomic DNA extraction followed the CTAB procedure and all individuals were genotyped using nine microsatellite loci (Cb3, Cb5, Cb6, Cb9, Cb11, Cb12, Cb13, Cb20, and Cb23) previously developed and optimized for *C. brasiliense* (Collevatti et al., 1999). For genotyping we followed the protocols described in Collevatti et al. (2010a). DNA fragments were sized in GS 3500 Genetic Analyzer (Applied Biosystems, CA, United States) using GeneScan ROX 500 size standard (Applied Biosystems, CA, United States), and the genotypes were obtained using GeneMapper v5.0 software (Applied Biosystems, CA, United States). We analyzed genotyping errors (allele dropout and null allele) using Micro-Checker 2.2.3 software (Van Oosterhout et al., 2004).

For node analysis, we estimated allelic richness based on rarefaction (*AR*; Mousadik and Petit, 1996), genetic diversity based on the expected heterozygosity under Hardy-Weinberg equilibrium (*He*; Nei, 1978) and inbreeding coefficient (*f*; Wright, 1951) for each sampling site. Analyses were performed using the package *hierfstat* (Goudet, 2005) implemented in R version 3.6.1 (R Core Team, 2019). For link analysis, we estimated the genetic differentiation among all pairs of sampling sites nested within landscapes using Wright’s *F*_*ST*_ (Weir and Cockerham, 1984), *G_*ST*_’* (Hedrick, 2005) and Jost’ *D* (Jost, 2008). Wright’s *F*_*ST*_ is a widely used parameter based on analysis of variance of allele frequencies, but is influenced by heterozygosity. *G_*ST*_’* is based on *F*_*ST*_, but takes into account the observed diversity within population and the number of subpopulations (Hedrick, 2005). Jost’ *D* is based on the effective number of alleles, but is unaffected by population size (Jost, 2008). Analyses were performed using the package *mmod* (Winter, 2012) also implemented in R version 3.6.1. We also estimated Slatkin’s *R*_*ST*_ and tested the hypothesis that *R*_*ST*_ = *F*_*ST*_ using the software Spagedi (Hardy and Vekemans, 2002).

Also, to verify differentiation among populations from different landscapes and within landscapes we performed an hierarchical AMOVA (analysis of molecular variance) implemented in the software Arlequin 3.5 (Excoffier and Lischer, 2010). We estimate the genetic differentiation among landscapes (*F*_*CT*_) and among populations within landscapes (*F*_*SC*_). Significance levels of 0.05 for each estimate were determined with 10,000 permutations. We also used Bayesian clustering simulations to assess the number of discrete genetic clusters (K) using the software Structure v. 2.3.4 (Pritchard et al., 2000), with admixture model of ancestry and correlated allele frequencies. We performed four independent runs for each K ranging from 1 to 10, to assess consistency of the results, with a burn-in period of 100,000 repetitions, followed by 1,000,000 Markov-Chain Monte Carlo (MCMC). To detect the number of K that best fits the data we used the ΔK method (Evanno et al., 2005) implemented in Structure Harvester v. 0.6.94 (Earl and von Holdt, 2012).

Finally, we estimated effective population size (*N*_*e*_) using the molecular co-ancestry method (Nomura, 2008) implemented in NeEstimator V2.1 (Do et al., 2014) to test the hypothesis that genetic variability depends on *N*_*e*_. Additionally, to test the hypothesis of loss of genetic diversity between generations (adults and juveniles), we performed two-sample t-Student tests for *He*, *AR, f* and *Ne.*

### Data Analysis

To analyze the effect of landscape composition and configuration, and effective population size on genetic variability and differentiation we first analyzed the scale of effect for each response variable (Jackson and Fahrig, 2012), using multiscale test of independence for multivariate vectors implemented in the *multifit* function (Huais, 2018), in R version 3.6.1 (R Core Team, 2019). We used linear models and R-squared (R^2^) as an index of the strength of the relationship (see the respective scale of effect to each variable in Supplementary Table 12).

Then, for the variables selected using the scale of effect (Supplementary Table 12), we analyzed the collinearity among the explanatory variables by estimating the variance inflation factor (VIF), which measures the inflation of the variance of a regression coefficient caused by multicollinearity in the model (Dormann et al., 2013). Analyses were performed using the *jtools* package (Long, 2019) in R version 3.6.1, with a stepwise approach to eliminate models with VIFs > 5.0 (Zuur et al., 2010). The analyses were performed for each response variable (see Supplementary Table 13).

To identify the effects of landscape on adaptive and neutral variation (see predictions in Figure 1), we then performed linear models using the explanatory variables selected by scale of effect and multicollinearity analysis (VIF ≤ 5.0, see Table 1). The response variables are continuous and we assumed Gaussian distributions on model fitting. We also built a null model by randomly sampling data keeping β equal to zero (constant variables) for all explanatory variables (absence of specific landscape processes). At node level we select the best predictive model based on Akaike Information Criteria (AIC). We estimated AIC corrected for small sample sizes (AICc), i.e., the difference of each model and the best model (ΔAICci), and Akaike’s Weight of Evidence (wAICc), i.e., the relative contribution of each model to explain the observed pattern, given a set of competing models (Burnham and Anderson, 2002). Models with ΔAICc < 2 were considered as equally plausible to explain the patterns (Zuur et al., 2009). For link level, we used models significance to select the best predictive model, because of the small sample size (five landscapes). All analyses were carried out using the packages *mgvc* (Wood, 2020), *bblme* (Bolker and R Development Core Team, 2017), *visreg* (Breheny and Burchett, 2017) and *ggplot2* (Wickham, 2016), available in R version 3.6.1 (R Core Team, 2019).

**TABLE 1:** Models performed at node and link levels for both neutral and adaptive quantitative traits measured in seeds and seedlings of *Caryocar brasiliense*.

**Analysis level**	**Response variable**	**Predictor variable**
**Node**	**Neutral genetic variability**	**Landscape – 500 m**
	Genetic diversity (*He*)	Composition heterogeneity (SHDI)
	Allele richness (*AR*)	**Genetic**
	Inbreeding coefficient (*f*)	Effective population size (*Ne*)
	**Adaptive quantitative variation**	**Landscape – 500 m**
	SLD	Composition heterogeneity (SHDI)
	SM	
	TG	**Genetic**
	LL	Effective population size (*N*_*e*_)
	RDM	
	ADM	
	**Evolvability**	**Landscape – 500 m**
	*CV*_*a*_%TG	Composition heterogeneity (SHDI)
	*CV*_*a*_%LL	
	*CV*_*a*_%RDM	**Genetic**
	*CV*_*a*_%ADM	Effective population size (*N*_*e*_)
**Link**	**Neutral genetic differentiation**	**Landscape – 2, 4, and 6 km**
	*F_*ST*__*Adults	Composition heterogeneity (SHDI) at 6 km
	*G_*ST*_’ _* Adults	Composition heterogeneity (SHDI) at 6 km
	Jost’D _ Adults	Composition heterogeneity (SHDI) at 6 km
	Inbreeding coefficient (*F*_*IS*_) _ Adults	Composition heterogeneity (SHDI) at 2 km
	*F*_*ST*_ _ Adults	Composition heterogeneity (SHDI) at 2 km
	*G_*ST*_’*_ Adults	Composition heterogeneity (SHDI) at 4 km
	Jost’D_ Adults	Composition heterogeneity (SHDI) at 4 km
	Inbreeding coefficient (*F*_*IS*_) _ Adults	Composition heterogeneity (SHDI) at 6 km
	**Adaptive quantitative differentiation**	**Landscape – 2, 4, and 6 km**
	*P*_*ST*_ – SLD	Composition heterogeneity (SHDI) at 6 km
	*P*_*ST*_ – SM	Composition heterogeneity (SHDI) at 4 km
	*Q*_*ST*_ – TG	Composition heterogeneity (SHDI) at 6 km
	*Q*_*ST*_ – LL	Composition heterogeneity (SHDI) at 6 km
	*Q*_*ST*_ – RDM	Composition heterogeneity (SHDI) at 6 km
	*Q*_*ST*_ – ADM	Composition heterogeneity (SHDI) at 2 km

*The genetic parameters are the average for each sampling site or landscape, or the additive genetic coefficient of variation (*CV*_*a*_%). SLD, seed longitudinal diameter (mm); SM, seed mass (mg); TG, time to seed germination; LL, leaf length (mm); RDM, root dry mass (g); ADM, aboveground dry mass (g).*

## Results

### Adaptive Quantitative Trait Variation

Additive genetic variance (*Va*) for seedling quantitative traits was high in most sampling sites (Supplementary Table 14), except for RDM, which show low values of *Va* in most sites. Narrow-sense heritability showed a wide range among sites (Supplementary Table 14), ranging from 0.003 to 1.000.

Genetic differentiation in quantitative traits (*Q*_*ST*_) was low in most landscapes (Supplementary Table 15), ranging from 0.000 to 0.230. Phenotypic differentiation in seed traits (*P*_*ST*_) was also low, ranging from 0.001 to 0.170 (Supplementary Table 15).

### Neutral Genetic Variation

Sampling sites had high genetic diversity (*He*) and allelic richness (*AR*) for both adults and juveniles (Supplementary Table 16) and did not differ between life stages (*He*, *t* = 0.776, *p* = 0.229; *AR*, *t* = −0.067, *p* = 0.642). The inbreeding coefficient (*f*) was low for both life stages (Supplementary Table 16) and also did not differ between them (*t* = −0.013, *p* = 0.849).

Overall, *F*_*ST*_ across landscapes was low, ranging from 0.005 to 0.022 for adults and 0.014 to 0.028 for juveniles (Supplementary Table 17). *G*_*ST*_’ ranged from 0.021 to 0.095 for adults and 0.061 to 0.170 for juveniles, and Jost’ *D* ranged from 0.018 to 0.093 for adults, and for 0.051 to 0.146 for juveniles (Supplementary Table 17). Slatkin’s *R*_*ST*_ for adults (*R*_*ST*_ = 0.027, SE = 0.002, *p* < 0.001) and juveniles (*R*_*ST*_ = 0.030, SE = 0.003, *p* < 0.001) did not differ from *F*_*ST*_ (adults, *p* = 0.293; juveniles, *p* = 0.370). Inbreeding coefficients within landscapes (*F*_*IS*_) was low for both adults and juveniles (Supplementary Table 17).

Genetic differentiation among landscapes was low for both adults (*F*_*CT*_ = 0.018, *p* = 0.001) and juveniles (*F*_*CT*_ = 0.011, *p* = 0.071). Differentiation between populations within landscapes was also low for both adults (*F*_*SC*_ = 0.012, *p* < 0.001) and juveniles (*F*_*SC*_ = 0.021, *p* < 0.001). Bayesian simulations showed a maximal value of L(D| K) with *K* = 2 for both adults (Supplementary Figure 1) and juveniles (Supplementary Figure 2). However, L(D| K) never reached a plateau and the values decreased and became more variable among runs, showing secondary peaks for *K* = 6 (Supplementary Figure 1), for adults, and *K* = 6 and *K* = 9 for juveniles. Coancestry plots also showed high admixture among populations (Supplementary Figure 2). All populations showed low effective population sizes (Supplementary Table 16), and *Ne* in adults was not different from juveniles (*t* = 0.346, *p* = 0.369).

### Landscape Effects on Adaptive Quantitative Traits

**FIGURE 3:**
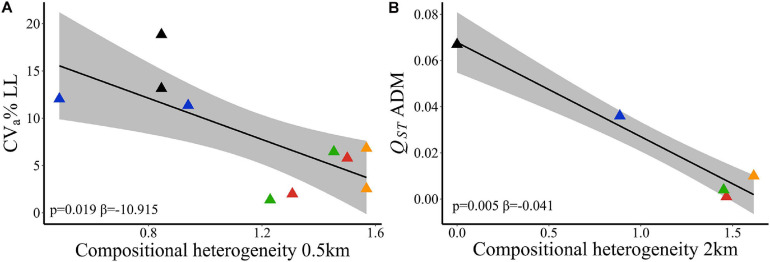
Relationships of adaptive quantitative traits and landscape compositional heterogeneity (SHDI) in *Caryocar brasiliense* based on 10 sampling sites in the Cerrado biome, Brazil. **(A)** Evolvability in leaf length (*CV*_*a*_% LL) and SHDI at node level. **(B)** Quantitative genetic differentiation in aboveground dry mass (*Q*_*ST*_ ADM) and SHDI at 2 km spatial scale. Black line is the linear regression fit and shaded area is the 95% confidence interval. Triangle color corresponds to the landscape: red = L1; blue = L2; orange = L3; green = L4; black = L5.

At node level using 500 m spatial scale, evolvability in leaf length (*CV*_*a*_% LL) was better explained by compositional heterogeneity (wAIC = 0.795, Table 2). Sites within landscapes with higher SHDI had lower LL evolvability (Figure 3A). Landscape features did not explain variation in adaptive traits among sites within landscapes. The null model was more likely than landscape compositional heterogeneity (SHDI) or effective population size (Supplementary Table 18). Effective population size (*Ne*) did not explain variation in any quantitative trait between sites within landscapes (Table 2). At link level, SHDI at 2 km spatial scale explained quantitative genetic differentiation between sites (*Q*_*ST*_) on ADM (*p* = 0.005, Supplementary Table 19). Landscapes with higher heterogeneity had lower aboveground dry mass (ADM) *Q*_*ST*_ (Figure 3B).

**TABLE 2:** Model selection to explain variation in neutral genetic diversity and adaptive quantitative traits variation in populations of *Caryocar brasiliense* in landscapes of the Brazilian Cerrado.

**Model**	***CV*_*a*_%** – **LL**		
	**AICc**	**ΔAICc**	**wAIC**
**Compositional heterogeneity (SHDI)**	**64.800**	**0.000**	**0.795**
Null model	67.800	3.000	0.177
Effective population size *(Ne)*	71.500	6.700	0.028

	**Adults** – ***AR***		
	**AICc**	**ΔAICc**	**wAIC**

**Compositional heterogeneity (SHDI)**	**10.500**	**0.000**	**0.960**
Null model	17.200	6.700	0.033
Effective population size *(Ne)*	20.500	10.000	0.007

	**Adults** – ***He***		
	**AICc**	**ΔAICc**	**wAIC**

**Compositional heterogeneity (SHDI)**	−**43.600**	**0.000**	**0.867**
Null model	−39.600	4.100	0.113
Effective population size *(Ne)*	−36.000	7.600	0.019

	**Juveniles – *AR***		
	**AICc**	**ΔAICc**	**wAIC**

**Compositional heterogeneity (SHDI)**	**20.000**	**0.000**	**0.490**
**Effective population size *(Ne)***	**20.800**	**0.800**	**0.330**
Null model	22.000	2.000	0.180

*LL, leaf length; *CV*_*a*_% – LL, additive genetic coefficient of variation of leaf length; *AR*, allelic richness; *He*, genetic diversity; *f*, inbreeding coefficient. AICc is the AIC corrected for sample size and number of parameters in the model; wAICc is the Akaike’s weight of evidence. Models with **Δ**AICc < 2.0 are in bold.*

### Landscape Effects on Neutral Genetic Variation

**FIGURE 4:**
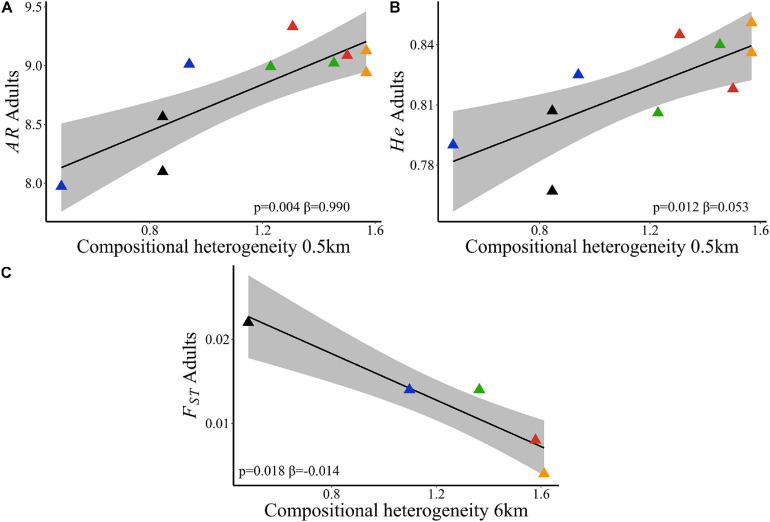
Relationships of neutral genetic variability and landscape compositional heterogeneity (SHDI) in *Caryocar brasiliense* based on 10 sampling sites in the Cerrado biome, Brazil. **(A)** Allelic richness (*AR*) in adults and SHDI at node level. **(B)** Genetic diversity (*He*) in adults and SHDI at node level. **(C)** Genetic differentiation (*F*_*ST*_) in adults and SHDI at 6 km spatial scale. Black line is the linear regression fit and shaded area is the 95% confidence interval. Triangle color corresponds to the landscape: red = L1; blue = L2; orange = L3; green = L4; black = L5.

**FIGURE 5:**
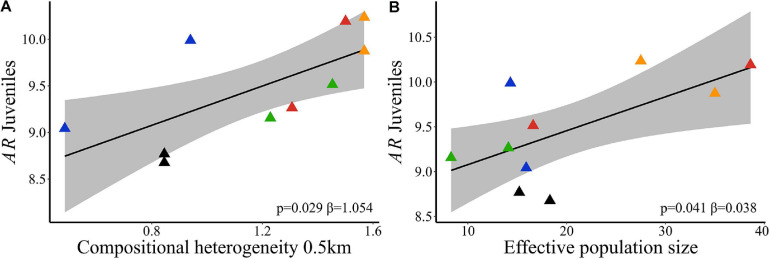
Relationships of neutral genetic variability and landscape compositional heterogeneity (SHDI) in *Caryocar brasiliense* based on 10 sampling sites in the Cerrado biome, Brazil. **(A)** Allelic richness (*AR*) in juveniles and SHDI at node level (%). **(B)** *AR* in juveniles and effective population size (*Ne*). Black line is the linear regression fit and shaded area is the 95% confidence interval. Triangle color corresponds to the landscape: red = L1; blue = L2; orange = L3; green = L4; black = L5.

Landscape compositional heterogeneity at 500 m spatial scale explained the variation observed in allelic richness (wAIC = 0.960, Table 2) and genetic diversity (wAIC = 0.867, Table 2) among adults. Sites in landscapes with higher compositional heterogeneity had higher *AR* (Figure 4A) and *He* (Figure 4B). *AR* in juveniles was also explained by SHDI (wAIC = 0.490, Table 2) and had positive effects (Figure 5A), and *Ne* (wAIC = 0.330, Table 2). Populations with higher *Ne* had higher *AR* (Figure 5B). Inbreeding (*f*) in adults and juveniles was not explained by any explanatory variables (Table 2). At link level, genetic differentiation in adults (*F_*ST*_)* was explained by SHDI at 6 km spatial scale (*p* = 0.018, Supplementary Table 19). Landscapes with higher SHDI tended to have lower *F*_*ST*_ (Figure 4C). *G_*ST*_’*, Jost’D and *F*_*IS*_ were not explained by our models (*p* > 0.10, Supplementary Table 19).

## Discussion

Our findings show increase of neutral genetic diversity and loss of adaptive genetic variation due to landscape changes in the savanna tree *C. brasiliense*. Landscape compositional heterogeneity was the best predictor of the response variables. For adaptive quantitative traits we found significant effects of landscape structure changes in leaf (LL) size, while for neutral loci landscape structure affected allelic richness (*AR*) and genetic diversity (*He*) on both adults and juveniles. However, different from our expectations, landscape metrics such as habitat cover (%) and functional connectivity could not explain neutral and adaptive genetic variation.

### Low Quality of Landscape Mosaic May Explain Decreased Evolutionary Potential of Adaptive Traits

Populations in sites with higher compositional heterogeneity tended to have lower evolutionary potential in leaf size (*CV*_*a*_% LL). Leaf size is correlated to plant water use efficiency, photosynthesis rate and resources retention (Westoby et al., 2002). Plants have the ability to respond to stress in several ways, including physiological and morphological modifications in leaves (Alpert and Simms, 2002; Rozendaal et al., 2006). In most of the studied landscapes (see Figure 2), the mosaics comprise mainly two types of anthropogenic matrices (agriculture and pasture) and intermingled by small and slim natural vegetation areas. Landscapes with low quality may be more instable and stressful for plants, leading to loss of variation in leaf traits and thus lower evolutionary potential, compared to landscapes with higher habitat cover (%) or more heterogeneous mosaic.

In Brazil, areas dominated by soybean, an important commodity, such as in our study area, farmers preserve only the minimum of natural vegetation required by environmental law. The environmental law requires the conservation of riparian forests (permanent preservation areas, PPAs) and a patch of natural vegetation with at least 20% of the farm area (called legal reserve). However, farmers usually allocate legal reserves in poor soil remnants that potentially provide low crop yields, and disconnected from riparian forests (PPAs), which may also contribute to the low-quality landscapes in intensive farming (Santos et al., 2020b). Therefore, variation in soil nutrient and moisture in these agricultural landscapes may be higher causing loss of variation in leaf size among sites, because of selection to fast growth in nutrient poor and dry areas, and relaxed selection in areas with higher nutrient inputs (for instance in edges of savanna and agriculture areas that are periodically fertilized), leading to lower evolutionary potential. The lower differentiation in aboveground dry mass (ADM *Q*_*ST*_) may also due to the loss of variation in leaf size among sites with a poor mosaic.

It is important to note that, although variation in some adaptive traits were not explained by changes in landscape structure (TG, DRM, and DSM), they have evolutionary potential to respond to environmental changes. However, some sites have very low additive genetic variance and thus evolutionary potential (*CV*_*a*_%), such as sites in the landscapes P3 and P4, implying limited capacity to respond to environmental changes, because evolutionary potential depends on population additive genetic variance (Houle et al., 2011). The lack of evolutionary potential may jeopardize species long-term persistence in these landscapes. In addition, *Q*_*ST*_ and *P*_*ST*_ were low in most landscapes, also showing lack of variation among sites and thus low potential to respond to selection and cope with environmental changes.

### Compositional Heterogeneity May Have Led to Increased Neutral Genetic Diversity by Fostering Pollen Dispersal

Our findings showed that adults in landscapes with higher compositional heterogeneity have higher allelic richness (*AR*) and genetic diversity (*He*), and juveniles have higher *AR*. For instance, populations in the protected area (L5), with the highest habitat cover (%) and structural connectivity and lower compositional heterogeneity showed the lowest values of genetic diversity and allelic richness, but the highest effective population size. On the other hand, landscape L3, with populations in a protected area, but with agroecosystem surrounding them, had higher genetic diversity and allelic richness than L5. We believe that the positive relationships between allelic richness and genetic diversity and compositional heterogeneity is related to pollen dispersal patterns.

Bats can potentially carry pollen over long distances because of their behavior and flight capacity (Bawa, 1990). However, *C. brasiliense* has mass-flowering with high synchrony of flowering of neighboring plants, leading to short-distance pollen dispersal (Collevatti et al., 2010a). In addition, *Glossophaga soricina*, the main *C. brasiliense* pollinator, tend to forage in groups remaining near the same patch of trees for a long time (Gribel and Hay, 1993). This behavior may cause high proportion of self-pollination and high probability of full-sibship within progeny arrays, because of the high density and clumped distribution of *C. brasiliense* (Collevatti et al., 2009, 2010a). Habitat loss may decrease population size and density in *C. brasiliense*, leading to lower number of individuals flowering within populations, decreasing the frequency of pollination between neighboring plants and increasing pollen dispersal distance (Collevatti et al., 2010a), and thus, genetic diversity. Heterozygous seedlings have higher survival probability in *C. brasiliense* (Collevatti and Hay, 2011), and self-seeds are more prone to abortion (Collevatti et al., 2009). Thus, mechanisms that foster gene flow by pollen and natural selection reducing the proportion of homozygous individuals may potentially increase genetic diversity and allelic richness.

Moreover, several mammals species with diverse body size and vagility disperse *C. brasiliense*’s seeds, and can carry-over seeds to different distances, and may respond differentially to landscape features. For instance, *C. brachyurus*, the maned wolf and *Mazama gouazoupira*, the brown brocket deer, are generalist species, with low sensitivity to landscape changes (Lyra-Jorge et al., 2010; Rodrigues et al., 2017), and may potentially promote *C. brasiliense’s* seed long-distance dispersal. Indeed, several Cerrado mammal species, especially generalists, can persist in fragmented landscapes (e.g., Lyra-Jorge et al., 2008, 2010; Lessa et al., 2012). These species can use heterogeneous landscapes that are mosaic of agricultural and patches of natural vegetation, maintaining landscape connectivity.

Abundance of trees in plant communities in Brazil tend to decline in landscapes with lower habitat amount (Rocha-Santos et al., 2017) and habitat loss may affect the genetic diversity and adaptive variation of Neotropical savanna tree species (Collevatti et al., 2020a). Indeed, our results suggest that a more diverse mosaic, composed of different types of land covers are increasing the gene flow and genetic diversity at large spatial scale. Particularly at the 6 km spatial scale, landscapes composed by different land covers may increase seed disperses and pollinators movement, or may provide additional habitat and resources for them (Ewers and Didham, 2006; Fahrig et al., 2011). The negative relationship between *F*_*ST*_ and compositional heterogeneity at 6 km spatial scale may be an evidence of this relationship. In fact, compositional heterogeneity seems to be an important factor shaping patterns of diversity in agricultural landscapes. For instance, landscape compositional heterogeneity increases plant diversity (e.g., Pardini et al., 2009), and bee diversity and richness in Atlantic Forest landscapes (Boscolo et al., 2017). Also, in landscapes in the same study area (LTER COFA) compositional heterogeneity increases plant richness and diversity (Santos et al., 2020b), and the evolutionary potential in adaptive quantitative traits in a bee pollinated and wind-dispersed savanna tree, *Tabebuia aurea* (Collevatti et al., 2020a). *Tabebuia aurea* was studied in the same landscapes in the LTER COFA project, and using the same experimental design as the present study (see Collevatti et al., 2020a), to compare the effects of landscape features in savanna tree species with different life history traits. As expected, the species responded to the landscape features, but in different ways. Neutral genetic diversity and evolutionary potential in populations of *C. brasiliense*, pollinated by bats and dispersed by terrestrial mammals, were affected mainly by landscape compositional heterogeneity, or mosaic quality, while habitat cover (%) explained better the variation in neutral genetic diversity and evolutionary potential in *T. aurea*, pollinated by large-sized bees and wind dispersed. *Glossophaga soricina*, the main *C. brasiliense*’s pollinator, is highly affected by the replacement of natural vegetation to agriculture and pasture, leading to a decrease in genetic diversity (Collevatti et al., 2020b), reinforcing the importance of mosaic quality to increase connectivity among savanna remnants and the effects of landscape in *C. brasiliense*.

Unexpectedly, our results show no time lag between adults and juveniles, despite the very recent agriculture expansion and fragmentation of the Cerrado biome in Central Brazil (∼60 years), when considering the life cycle of the species. We found no difference in genetic diversity between adults and juveniles and no effects of habitat loss on genetic diversity, but a positive effect of landscape heterogeneity on both, despite lower habitat amount. The lack of a time lag is most likely due to the mosaic of different types of ecosystems, such as seasonally dry and riparian forests, savannas, wetlands and agroecosystems increasing compositional heterogeneity and favoring gene flow. We also found low genetic differentiation among populations from different landscapes and high admixture for both adults and juveniles. This results was expected since our analyses encompass populations at a regional spatial scale, that may have diverged only recently.

It is also important to note that populations in savanna remnants in landscapes outside protected areas (L1, L2, and L4), had slightly higher neutral and adaptive genetic variation than in savannas in protected areas (L3 and L5). Indeed, L1, L2, and L4 are in farm legal reserves, i.e., remnants of vegetation preserved in farms in compliance with Brazilian environmental law. Recently, Brazilian senators proposed to omit the obligation of legal reserves from environmental law, which may jeopardize the conservation of biodiversity and ecosystem services in Brazil (Metzger et al., 2019). Our results and a previous study in the same landscapes with *Tabebuia aurea* (Collevatti et al., 2020a) reinforce the importance of maintaining the legal reserves, to conserve genetic diversity and gene flow among populations.

### Implications for Policymakers

Our results suggest that management strategies improving landscape and legal reserves quality are critical for the persistence of *C. brasiliense* in intensive farming landscapes. Except for the protected areas, natural vegetation remnants in the landscapes are legal reserves. Therefore, any initiative to ignore the conservation of these areas will compromise the biodiversity in the study area. In contrast, actions to exceed environmental law compliance are necessary to avoid loss of evolutionary potential in areas of intensive crop cultivation.

The natural mosaic established by the relief conditions in the study area helps maintain the patches of natural vegetation in some landscapes. Therefore, strategies to improve agricultural landscape quality – particularly those dominated by commodities such as soybean – are critical for genetic diversity conservation of tree species. Our results also highlights that strategies improving mosaic quality must be implemented at local scale, since we found composition heterogeneity effects at small scales such as 500 m. Better practices in agroecosystems management, such as the restoration of degraded pastures, savanna and forest remnants may increase the mosaic heterogeneity and quality increasing connectivity (Donald and Evans, 2006; Santos et al., 2020a) and genetic variation.

## Concluding Remarks

In conclusion, we found that compositional heterogeneity in agriculture landscapes is the most important landscape characteristic shaping neutral and adaptive quantitative trait variation in *C. brasiliense* in our study area. However, the response of adaptive quantitative traits variability to landscape changes was different from neutral variation. On one hand, high compositional heterogeneity is associated to the loss of evolvability in adaptive trait, contrary to our hypothesis (v). On the other hand, it is associated to higher neutral variability and genetic differentiation among populations, corroborating our initial hypotheses (i and iii) We also found no evidence of time lag in the response of *C. brasiliense* to landscape changes (hypothesis vi), showing that, despite the recent fragmentation of the Cerrado biome, the genetic variability in parental generations (adults) is already affected by ongoing landscape changes, which may cause a cascate effect in the next generations.

## Data Availability Statement

The data and additional supporting information may be found in the online version of this article as Supplementary Material.

## Author Contributions

RC and MR conceived and funded the work. TA and FR obtained the data. RC, JS, MR, and LC designed the experiment, field sampling, and statistical analyses. TA, JS, and MP carried out analyses. RC, TA, and MP wrote the original draft. All authors contributed to the manuscript and approved the final version.

## Conflict of Interest

The authors declare that the research was conducted in the absence of any commercial or financial relationships that could be construed as a potential conflict of interest.
