# Supplementary material for: Agricultural Landscape Heterogeneity Matter: Responses of Neutral Genetic Diversity and Adaptive Traits in a Neotropical Savanna Tree
Source: Front Genet. 2021 Feb 4;11:606222. doi: 10.3389/fgene.2020.606222 (PMC7890196; doi:10.3389/fgene.2020.606222)
Supplement: Supplementary file 1 [file Data_Sheet_1.docx]

**Agricultural landscape heterogeneity matters: responses of neutral genetic diversity and adaptive traits in a Neotropical savanna tree**

Tatiana Souza do Amaral, Juliana Silveira dos Santos, Fernanda Fraga Rosa, Marcelo Bruno Pessôa, Lázaro José Chaves, Milton Cezar Ribeiro, Rosane Garcia Collevatti

**Appendix S1 - Tables**

**Table S1**. Sampling location for the five landscapes and 10 sites of *Caryocar brasiliense* in the Cerrado biome, Brazil. Coordinates are in decimal degree. L3F5 and L3F6 are sampling sites in the same large savanna remnant in a protected area (National Forest of Silvânia), as well as L5F9 and L5F10 (State Park of Caldas Novas). Percentage of savanna cover is based on 2 km buffer.

| **Landscape** | **Local** | **Savanna cover (%)** | **Site** | **Altitude (m)** | **Distance between focal sites (m)** | **Coordinates** | |
| --- | --- | --- | --- | --- | --- | --- | --- |
|  |  |  |  |  |  | **Lat** | **Long** |
|  |  |  |  |  |  |  |  |
| L1 | Leopoldo de Bulhões | 29% | L1F1 | 1058 | 2383 | -16.6067 | -48.7631 |
|  |  |  | L1F2 | 1061 |  | -16.5933 | -48.7809 |
| L2 | Vianópolis | 23% | L2F3 | 985 | 1494 | -16.8290 | -48.5154 |
|  |  |  | L2F4 | 969 |  | -16.8166 | -48.5127 |
| L3 | National Forest of Silvania | 35% | L3F5 | 945 | 1743 | -16.6358 | -48.6501 |
|  |  |  | L3F6 | 972 |  | -16.6414 | -48.6660 |
| L4 | Silvânia | 15% | L4F7 | 1022 | 2194 | -16.7108 | -48.7949 |
|  |  |  | L4F8 | 1030 |  | -16.6919 | -48.7970 |
| L5 | State Park of Caldas Novas | 100% | L5F9 | 1030 | 2601 | -17.7769 | -48.6890 |
|  |  |  | L5F10 | 981.5 |  | -17.7769 | -48.7145 |

**Table S2.** Landscape metrics measured at node level, in buffers of 0.5 km drew around 10 sampling sites of *Caryocar brasiliense*, in the Cerrado biome, Brazil. HA, habitat (savanna) amount (%); SHDI, compositional heterogeneity; FC, functional connectivity (hectares); Sa+Se+Ri, amount of savanna+seasonal forest+riparian forest (%); LQ_GLS = landscape quality for *Glossophaga soricina*; LQ_TAT, landscape quality for *Tapirus terrestris*; LQ_DA, landscape quality for *Dasyprocta* sp.; LQ_CHB, landscape quality for *Chrysocyon brachyurus*; LQ_MA, landscape quality for *Mazama americana* and *M. guazoupira*, LQ_PCA, landscape quality using the PCA resistance value calculated for the pollinator and dispersers of *C. brasiliense*.

| **Site** | **HA** | **SHDI** | **FC** | **Sa+Se+Ri** | **LQ_GLS** | **LQ_TAT** | **LQ_DA** | **LQ_CHB** | **LQ_MA** | **LQ_PCA** |
| --- | --- | --- | --- | --- | --- | --- | --- | --- | --- | --- |
| **L1F1** | 36.306 | 1.501 | 781.130 | 49.530 | 0.572 | 0.742 | 0.742 | 0.784 | 0.881 | 0.792 |
| **L1F2** | 13.019 | 1.307 | 169.300 | 16.356 | 0.315 | 0.663 | 0.691 | 0.855 | 0.881 | 0.756 |
| **L2F3** | 16.219 | 0.486 | 540.880 | 16.965 | 0.235 | 0.651 | 0.651 | 0.894 | 0.899 | 0.754 |
| **L2F4** | 31.965 | 0.940 | 191.650 | 36.424 | 0.396 | 0.705 | 0.709 | 0.855 | 0.885 | 0.776 |
| **L3F5** | 39.184 | 1.568 | 986.200 | 65.040 | 0.662 | 0.791 | 0.791 | 0.680 | 0.871 | 0.796 |
| **L3F6** | 39.184 | 1.568 | 986.200 | 65.040 | 0.662 | 0.791 | 0.791 | 0.680 | 0.871 | 0.796 |
| **L4F7** | 14.310 | 1.229 | 154.030 | 24.823 | 0.334 | 0.682 | 0.688 | 0.806 | 0.890 | 0.755 |
| **L4F8** | 17.811 | 1.453 | 315.250 | 27.529 | 0.414 | 0.687 | 0.691 | 0.814 | 0.891 | 0.768 |
| **L5F9** | 76.704 | 0.846 | 18206.000 | 82.002 | 0.829 | 0.825 | 0.825 | 0.833 | 0.863 | 0.843 |
| **L5F10** | 76.704 | 0.846 | 18206.000 | 82.002 | 0.829 | 0.825 | 0.825 | 0.833 | 0.863 | 0.843 |

**Table S3.** Landscape metrics measured at the link level, in buffers of 2km, 4km and 6km in sites of *Caryocar brasiliense*, in the Cerrado biome, Brazil. Sa+Se+Ri = amount of savanna+seasonal forest+riparian forest (ha); HA = habitat (savanna) amount (%); SHDI, compositional heterogeneity; FC, functional connectivity (hectares); LQ_GLS, landscape quality for *Glossophaga soricina*; LQ_TAT, landscape quality for *Tapirus terrestris*; LQ_DA, landscape quality for *Dasyprocta* sp.; LQ_CHB, landscape quality for *Chrysocyon brachyurus*; LQ_MA, landscape quality for *Mazama americana* and *M. guazoupira*, LQ_PCA, landscape quality using the PCA resistance value calculated for the pollinator and dispersers of *C. brasiliense*.

| **Scale** | **Landscape** | **Sa+Se+Ri** | **HA** | **SHDI** | **FC** | | **LQ_ GLS** | | **LQ_TAT** | **LQ_DA** | **LQ_CHB** | **LQ_MA** | **LQ_PCA** |
| --- | --- | --- | --- | --- | --- | --- | --- | --- | --- | --- | --- | --- | --- |
| **2 km** | **L1** | 547.963 | 28.958 | 1.466 | 408140.000 | | | 0.512 | 0.731 | 0.731 | 0.780 | 0.888 | 0.787 |
|  | **L2** | 336.073 | 23.301 | 0.886 | 333922.000 | | | 0.325 | 0.678 | 0.681 | 0.867 | 0.890 | 0.765 |
|  | **L3** | 731.268 | 33.836 | 1.615 | 431704.000 | | | 0.628 | 0.770 | 0.768 | 0.690 | 0.870 | 0.788 |
|  | **L4** | 338.965 | 15.402 | 1.452 | 378859.000 | | | 0.408 | 0.685 | 0.686 | 0.800 | 0.892 | 0.765 |
|  | **L5** | 1256.550 | 100.000 | 0.000 | 600321.000 | | | 0.900 | 0.900 | 0.900 | 0.900 | 0.900 | 0.900 |
| **4 km** | **L1** | 1407.660 | 11.157 | 1.611 | 902784.000 | | | 0.432 | 0.673 | 0.673 | 0.739 | 0.865 | 0.743 |
|  | **L2** | 976.528 | 8.450 | 1.061 | 769658.000 | | | 0.289 | 0.657 | 0.656 | 0.806 | 0.887 | 0.744 |
|  | **L3** | 1656.458 | 15.022 | 1.548 | 970983.000 | | | 0.442 | 0.698 | 0.693 | 0.738 | 0.877 | 0.756 |
|  | **L4** | 1149.420 | 8.885 | 1.389 | 982315.000 | | | 0.406 | 0.670 | 0.670 | 0.784 | 0.893 | 0.759 |
|  | **L5** | 5026.493 | 100.000 | 0.000 | 2245905.000 | | | 0.900 | 0.900 | 0.900 | 0.900 | 0.900 | 0.900 |
| **6 km** | **L1** | 3206.983 | 8.472 | 1.579 | 1605608.000 | | | 0.450 | 0.677 | 0.676 | 0.722 | 0.872 | 0.747 |
|  | **L2** | 2082.278 | 6.035 | 1.098 | 1260531.000 | | | 0.285 | 0.654 | 0.654 | 0.792 | 0.884 | 0.739 |
|  | **L3** | 3297.423 | 10.509 | 1.612 | 2066757.000 | | | 0.439 | 0.683 | 0.677 | 0.722 | 0.863 | 0.744 |
|  | **L4** | 2287.705 | 5.401 | 1.364 | 1477864.000 | | | 0.401 | 0.662 | 0.661 | 0.776 | 0.891 | 0.754 |
|  | **L5** | 10374.065 | 89.128 | 0.482 | 4677359.000 | | | 0.870 | 0.859 | 0.859 | 0.860 | 0.873 | 0.866 |
| **4-2km** | **L1** | 859.698 |  |  |  |  | | |  |  |  |  |  |
|  | **L2** | 640.455 |  |  |  |  | | |  |  |  |  |  |
|  | **L3** | 925.190 |  |  |  |  | | |  |  |  |  |  |
|  | **L4** | 810.455 |  |  |  |  | | |  |  |  |  |  |
|  | **L5** | 3769.943 |  |  |  |  | | |  |  |  |  |  |
| **6-4km** | **L1** | 1799.323 |  |  |  |  | | |  |  |  |  |  |
|  | **L2** | 1105.750 |  |  |  |  | | |  |  |  |  |  |
|  | **L3** | 1640.965 |  |  |  |  | | |  |  |  |  |  |
|  | **L4** | 1138.285 |  |  |  |  | | |  |  |  |  |  |
|  | **L5** | 5347.573 |  |  |  |  | | |  |  |  |  |  |

**Table S4**. Percentage of each land cover class (Pi), calculated based on buffers of 0.5 km drew around each sampling site, and quality scores (Qi) for pollinators and seed dispersers of *Caryocar brasiliense* for each land cover class, based on experts opinion. GLS, *Glossophaga soricina*; TAT, *Tapirus terrestris*; MA, *Mazama americana* and *M. guazoupira*; DA, *Dasyprocta* sp.; CHB, *Chrysocyon brachyurus*; PCA, PCA resistance value calculated for the pollinator and dispersers of *C. brasiliense*.

| **Site** | | **agriculture_Pi** | **pasture_Pi** | **riparian_ forest_Pi** | **roads_Pi** | **rural_build_Pi** | | **savana_Pi** | **seasonal_ forest_Pi** | **eucalyptus_Pi** | **urban_area_Pi** | **water_Pi** | **wetland_Pi** |
| --- | --- | --- | --- | --- | --- | --- | --- | --- | --- | --- | --- | --- | --- |
| **L1F1** | | 30.444 | 18.000 | 5.762 | 0.194 | 0.083 | | 36.306 | 7.462 | 0.475 | 1.104 | 0.170 | 0.000 |
| **L1F2** | | 56.101 | 7.640 | 2.001 | 1.943 | 0.000 | | 13.019 | 1.336 | 17.960 | 0.000 | 0.000 | 0.000 |
| **L2F3** | | 83.035 | 0.000 | 0.746 | 0.000 | 0.000 | | 16.219 | 0.000 | 0.000 | 0.000 | 0.000 | 0.000 |
| **L2F4** | | 60.527 | 0.000 | 4.459 | 0.266 | 0.870 | | 31.965 | 0.000 | 1.915 | 0.000 | 0.000 | 0.000 |
| **L3F5** | | 19.602 | 12.855 | 19.036 | 0.000 | 1.026 | | 39.184 | 6.820 | 1.144 | 0.000 | 0.073 | 0.261 |
| **L3F6** | | 19.602 | 12.855 | 19.036 | 0.000 | 1.026 | | 39.184 | 6.820 | 1.144 | 0.000 | 0.073 | 0.261 |
| **L4F7** | | 62.358 | 5.112 | 8.102 | 0.000 | 0.071 | | 14.310 | 2.410 | 7.438 | 0.000 | 0.199 | 0.000 |
| **L4F8** | | 46.109 | 20.855 | 6.589 | 0.000 | 0.182 | | 17.811 | 3.129 | 5.175 | 0.000 | 0.151 | 0.000 |
| **L5F9** | | 0.500 | 13.663 | 3.534 | 0.144 | 0.222 | | 76.704 | 1.599 | 0.005 | 3.259 | 0.097 | 0.072 |
| **L5F10** | | 0.500 | 13.663 | 3.534 | 0.144 | 0.222 | | 76.704 | 1.599 | 0.005 | 3.259 | 0.097 | 0.072 |
|  | |  |  |  |  |  | |  |  |  |  |  |  |
| **Site** | | **Qi_agriculture_GLS** | **Qi_pasture_GLS** | **Qi_riparian_forest_GLS** | **Qi_roads_GLS** | **Qi_rural_build_GLS** | | **Qi_savana_GLS** | **Qi_seasonal_forest_GLS** | **Qi_tree_plantation_GLS** | **Qi_urban_area_GLS** | **Qi_water_GLS** | **Qi_wet_grassland_GLS** |
| **L1F1** | | 0.900 | 0.500 | 0.200 | 0.800 | 0.500 | | 0.100 | 0.100 | 0.600 | 0.300 | 0.500 | 0.400 |
| **L1F2** | | 0.900 | 0.500 | 0.200 | 0.800 | 0.500 | | 0.100 | 0.100 | 0.600 | 0.300 | 0.500 | 0.400 |
| **L2F3** | | 0.900 | 0.500 | 0.200 | 0.800 | 0.500 | | 0.100 | 0.100 | 0.600 | 0.300 | 0.500 | 0.400 |
| **L2F4** | | 0.900 | 0.500 | 0.200 | 0.800 | 0.500 | | 0.100 | 0.100 | 0.600 | 0.300 | 0.500 | 0.400 |
| **L3F5** | | 0.900 | 0.500 | 0.200 | 0.800 | 0.500 | | 0.100 | 0.100 | 0.600 | 0.300 | 0.500 | 0.400 |
| **L3F6** | | 0.900 | 0.500 | 0.200 | 0.800 | 0.500 | | 0.100 | 0.100 | 0.600 | 0.300 | 0.500 | 0.400 |
| **L4F7** | | 0.900 | 0.500 | 0.200 | 0.800 | 0.500 | | 0.100 | 0.100 | 0.600 | 0.300 | 0.500 | 0.400 |
| **L4F8** | | 0.900 | 0.500 | 0.200 | 0.800 | 0.500 | | 0.100 | 0.100 | 0.600 | 0.300 | 0.500 | 0.400 |
| **L5F9** | | 0.900 | 0.500 | 0.200 | 0.800 | 0.500 | | 0.100 | 0.100 | 0.600 | 0.300 | 0.500 | 0.400 |
| **L5F10** | | 0.900 | 0.500 | 0.200 | 0.800 | 0.500 | | 0.100 | 0.100 | 0.600 | 0.300 | 0.500 | 0.400 |
|  | |  |  |  |  |  | |  |  |  |  |  |  |
| **Site** | | **Qi_agriculture_TAT** | **Qi_pasture_TAT** | **Qi_riparian_forest_TAT** | **Qi_roads_TAT** | **Qi_rural_build_TAT** | | **Qi_savana_TAT** | **Qi_seasonal_forest_TAT** | **Qi_tree_plantation_TAT** | **Qi_urban_area_TAT** | **Qi_water_TAT** | **Qi_wet_grassland_TAT** |
| **L1F1** | | 0.400 | 0.400 | 0.100 | 0.600 | 1.000 | | 0.100 | 0.100 | 0.300 | 1.000 | 0.100 | 0.100 |
| **L1F2** | | 0.400 | 0.400 | 0.100 | 0.600 | 1.000 | | 0.100 | 0.100 | 0.300 | 1.000 | 0.100 | 0.100 |
| **L2F3** | | 0.400 | 0.400 | 0.100 | 0.600 | 1.000 | | 0.100 | 0.100 | 0.300 | 1.000 | 0.100 | 0.100 |
| **L2F4** | | 0.400 | 0.400 | 0.100 | 0.600 | 1.000 | | 0.100 | 0.100 | 0.300 | 1.000 | 0.100 | 0.100 |
| **L3F5** | | 0.400 | 0.400 | 0.100 | 0.600 | 1.000 | | 0.100 | 0.100 | 0.300 | 1.000 | 0.100 | 0.100 |
| **L3F6** | | 0.400 | 0.400 | 0.100 | 0.600 | 1.000 | | 0.100 | 0.100 | 0.300 | 1.000 | 0.100 | 0.100 |
| **L4F7** | | 0.400 | 0.400 | 0.100 | 0.600 | 1.000 | | 0.100 | 0.100 | 0.300 | 1.000 | 0.100 | 0.100 |
| **L4F8** | | 0.400 | 0.400 | 0.100 | 0.600 | 1.000 | | 0.100 | 0.100 | 0.300 | 1.000 | 0.100 | 0.100 |
| **L5F9** | | 0.400 | 0.400 | 0.100 | 0.600 | 1.000 | | 0.100 | 0.100 | 0.300 | 1.000 | 0.100 | 0.100 |
| **L5F10** | | 0.400 | 0.400 | 0.100 | 0.600 | 1.000 | | 0.100 | 0.100 | 0.300 | 1.000 | 0.100 | 0.100 |
|  | |  |  |  |  |  | |  |  |  |  |  |  |
| **Site** | | **Qi_agriculture_MA** | **Qi_pasture_MA** | **Qi_riparian_forest_MA** | **Qi_roads_MA** | **Qi_rural_build_MA** | | **Qi_savana_MA** | **Qi_seasonal_forest_MA** | **Qi_tree_plantation_MA** | **Qi_urban_area_MA** | **Qi_water_MA** | **Qi_wet_grassland_MA** |
| **L1F1** | | 0.100 | 0.100 | 0.200 | 1.000 | 1.000 | | 0.100 | 0.100 | 0.100 | 1.000 | 0.500 | 0.400 |
| **L1F2** | | 0.100 | 0.100 | 0.200 | 1.000 | 1.000 | | 0.100 | 0.100 | 0.100 | 1.000 | 0.500 | 0.400 |
| **L2F3** | | 0.100 | 0.100 | 0.200 | 1.000 | 1.000 | | 0.100 | 0.100 | 0.100 | 1.000 | 0.500 | 0.400 |
| **L2F4** | | 0.100 | 0.100 | 0.200 | 1.000 | 1.000 | | 0.100 | 0.100 | 0.100 | 1.000 | 0.500 | 0.400 |
| **L3F5** | | 0.100 | 0.100 | 0.200 | 1.000 | 1.000 | | 0.100 | 0.100 | 0.100 | 1.000 | 0.500 | 0.400 |
| **L3F6** | | 0.100 | 0.100 | 0.200 | 1.000 | 1.000 | | 0.100 | 0.100 | 0.100 | 1.000 | 0.500 | 0.400 |
| **L4F7** | | 0.100 | 0.100 | 0.200 | 1.000 | 1.000 | | 0.100 | 0.100 | 0.100 | 1.000 | 0.500 | 0.400 |
| **L4F8** | | 0.100 | 0.100 | 0.200 | 1.000 | 1.000 | | 0.100 | 0.100 | 0.100 | 1.000 | 0.500 | 0.400 |
| **L5F9** | | 0.100 | 0.100 | 0.200 | 1.000 | 1.000 | | 0.100 | 0.100 | 0.100 | 1.000 | 0.500 | 0.400 |
| **L5F10** | | 0.100 | 0.100 | 0.200 | 1.000 | 1.000 | | 0.100 | 0.100 | 0.100 | 1.000 | 0.500 | 0.400 |
|  | |  |  |  |  |  | |  |  |  |  |  |  |
| **Site** | | **Qi_agriculture_DA** | **Qi_pasture_DA** | **Qi_riparian_forest_DA** | **Qi_roads_DA** | **Qi_rural_build_DA** | | **Qi_savana_DA** | **Qi_seasonal_forest_DA** | **Qi_tree_plantation_DA** | **Qi_urban_area_DA** | **Qi_water_DA** | **Qi_wet_grassland_DA** |
| **L1F1** | | 0.400 | 0.400 | 0.100 | 0.100 | 1.000 | | 0.100 | 0.100 | 0.200 | 1.000 | 1.000 | 0.500 |
| **L1F2** | | 0.400 | 0.400 | 0.100 | 0.100 | 1.000 | | 0.100 | 0.100 | 0.200 | 1.000 | 1.000 | 0.500 |
| **L2F3** | | 0.400 | 0.400 | 0.100 | 0.100 | 1.000 | | 0.100 | 0.100 | 0.200 | 1.000 | 1.000 | 0.500 |
| **L2F4** | | 0.400 | 0.400 | 0.100 | 0.100 | 1.000 | | 0.100 | 0.100 | 0.200 | 1.000 | 1.000 | 0.500 |
| **L3F5** | | 0.400 | 0.400 | 0.100 | 0.100 | 1.000 | | 0.100 | 0.100 | 0.200 | 1.000 | 1.000 | 0.500 |
| **L3F6** | | 0.400 | 0.400 | 0.100 | 0.100 | 1.000 | | 0.100 | 0.100 | 0.200 | 1.000 | 1.000 | 0.500 |
| **L4F7** | | 0.400 | 0.400 | 0.100 | 0.100 | 1.000 | | 0.100 | 0.100 | 0.200 | 1.000 | 1.000 | 0.500 |
| **L4F8** | | 0.400 | 0.400 | 0.100 | 0.100 | 1.000 | | 0.100 | 0.100 | 0.200 | 1.000 | 1.000 | 0.500 |
| **L5F9** | | 0.400 | 0.400 | 0.100 | 0.100 | 1.000 | | 0.100 | 0.100 | 0.200 | 1.000 | 1.000 | 0.500 |
| **L5F10** | | 0.400 | 0.400 | 0.100 | 0.100 | 1.000 | | 0.100 | 0.100 | 0.200 | 1.000 | 1.000 | 0.500 |
|  | |  |  |  |  |  | |  |  |  |  |  |  |
| **Site** | | **Qi_agriculture_CHB** | **Qi_pasture_CHB** | **Qi_riparian_forest_CHB** | **Qi_roads_CHB** | **Qi_rural_build_CHB** | | **Qi_savana_CHB** | **Qi_seasonal_forest_CHB** | **Qi_tree_plantation_CHB** | **Qi_urban_area_CHB** | **Qi_water_CHB** | **Qi_wet_grassland_CHB** |
| **L1F1** | | 0.100 | 0.100 | 0.900 | 0.100 | 1.000 | | 0.100 | 0.900 | 0.200 | 0.800 | 1.000 | 0.800 |
| **L1F2** | | 0.100 | 0.100 | 0.900 | 0.100 | 1.000 | | 0.100 | 0.900 | 0.200 | 0.800 | 1.000 | 0.800 |
| **L2F3** | | 0.100 | 0.100 | 0.900 | 0.100 | 1.000 | | 0.100 | 0.900 | 0.200 | 0.800 | 1.000 | 0.800 |
| **L2F4** | | 0.100 | 0.100 | 0.900 | 0.100 | 1.000 | | 0.100 | 0.900 | 0.200 | 0.800 | 1.000 | 0.800 |
| **L3F5** | | 0.100 | 0.100 | 0.900 | 0.100 | 1.000 | | 0.100 | 0.900 | 0.200 | 0.800 | 1.000 | 0.800 |
| **L3F6** | | 0.100 | 0.100 | 0.900 | 0.100 | 1.000 | | 0.100 | 0.900 | 0.200 | 0.800 | 1.000 | 0.800 |
| **L4F7** | | 0.100 | 0.100 | 0.900 | 0.100 | 1.000 | | 0.100 | 0.900 | 0.200 | 0.800 | 1.000 | 0.800 |
| **L4F8** | | 0.100 | 0.100 | 0.900 | 0.100 | 1.000 | | 0.100 | 0.900 | 0.200 | 0.800 | 1.000 | 0.800 |
| **L5F9** | | 0.100 | 0.100 | 0.900 | 0.100 | 1.000 | | 0.100 | 0.900 | 0.200 | 0.800 | 1.000 | 0.800 |
| **L5F10** | | 0.100 | 0.100 | 0.900 | 0.100 | 1.000 | | 0.100 | 0.900 | 0.200 | 0.800 | 1.000 | 0.800 |
|  | |  |  |  |  |  | |  |  |  |  |  |  |
| **Site** | **Qi_agriculture_pca** | | **Qi_pasture_pca** | **Qi_riparian_pca** | **Qi_roads_pca** | **Qi_rural_pca** | **Qi_savana_pca** | | **Qi_seasonal_pca** | **Qi_tree_pca** | **Qi_urban_pca** | **Qi_water_pca** | **Qi_wet_pca** |
| **L1F1** | | 0.274 | 0.227 | 0.267 | 0.328 | 1.000 | | 0.100 | 0.233 | 0.254 | 0.955 | 0.625 | 0.426 |
| **L1F2** | | 0.274 | 0.227 | 0.267 | 0.328 | 1.000 | | 0.100 | 0.233 | 0.254 | 0.955 | 0.625 | 0.426 |
| **L2F3** | | 0.274 | 0.227 | 0.267 | 0.328 | 1.000 | | 0.100 | 0.233 | 0.254 | 0.955 | 0.625 | 0.426 |
| **L2F4** | | 0.274 | 0.227 | 0.267 | 0.328 | 1.000 | | 0.100 | 0.233 | 0.254 | 0.955 | 0.625 | 0.426 |
| **L3F5** | | 0.274 | 0.227 | 0.267 | 0.328 | 1.000 | | 0.100 | 0.233 | 0.254 | 0.955 | 0.625 | 0.426 |
| **L3F6** | | 0.274 | 0.227 | 0.267 | 0.328 | 1.000 | | 0.100 | 0.233 | 0.254 | 0.955 | 0.625 | 0.426 |
| **L4F7** | | 0.274 | 0.227 | 0.267 | 0.328 | 1.000 | | 0.100 | 0.233 | 0.254 | 0.955 | 0.625 | 0.426 |
| **L4F8** | | 0.274 | 0.227 | 0.267 | 0.328 | 1.000 | | 0.100 | 0.233 | 0.254 | 0.955 | 0.625 | 0.426 |
| **L5F9** | | 0.274 | 0.227 | 0.267 | 0.328 | 1.000 | | 0.100 | 0.233 | 0.254 | 0.955 | 0.625 | 0.426 |
| **L5F10** | | 0.274 | 0.227 | 0.267 | 0.328 | 1.000 | | 0.100 | 0.233 | 0.254 | 0.955 | 0.625 | 0.426 |

**Table S5**. Percentage of each land cover class (Pi), calculated based on buffers of 2km, 4km and 6km for five landscapes, and quality scores (Qi) for pollinators and seed dispersers of *Caryocar brasiliense* for each land cover class, based on expert’s opinion. GLS, *Glossophaga soricina*; TAT, *Tapirus terrestris*; MA, *Mazama americana* and *M. guazoupira*; DA, *Dasyprocta* sp.; CHB, *Chrysocyon brachyurus*; PCA, PCA resistance value calculated for the pollinator and dispersers of *C. brasiliense*.

| **Scale** | **Landscape** | **agriculture_Pi** | **pasture_Pi** | **riparian_ forest_Pi** | **roads_Pi** | **rural_build_Pi** | **savana_Pi** | **seasonal_ forest_Pi** | **tree_plantation_Pi** | **urban_area_Pi** | **water_Pi** | **wetland_Pi** |
| --- | --- | --- | --- | --- | --- | --- | --- | --- | --- | --- | --- | --- |
| **2km** | **L1** | 38.550 | 16.870 | 6.410 | 0.430 | 0.060 | 28.960 | 8.240 | 0.260 | 0.000 | 0.170 | 0.050 |
|  | **L2** | 69.260 | 2.220 | 3.380 | 0.300 | 0.440 | 23.300 | 0.060 | 1.040 | 0.000 | 0.000 | 0.000 |
|  | **L3** | 21.640 | 17.660 | 17.290 | 0.000 | 1.200 | 33.840 | 7.070 | 0.850 | 0.000 | 0.080 | 0.380 |
|  | **L4** | 47.610 | 21.210 | 5.830 | 0.000 | 0.130 | 15.400 | 5.740 | 3.790 | 0.000 | 0.210 | 0.080 |
|  | **L5** | 0.000 | 0.000 | 0.000 | 0.000 | 0.000 | 100.000 | 0.000 | 0.000 | 0.000 | 0.000 | 0.000 |
| **4km** | **L1** | 43.240 | 22.920 | 6.730 | 0.440 | 1.020 | 11.160 | 10.110 | 2.400 | 1.390 | 0.450 | 0.140 |
|  | **L2** | 70.420 | 8.920 | 6.530 | 0.170 | 0.390 | 8.450 | 4.450 | 0.310 | 0.000 | 0.070 | 0.290 |
|  | **L3** | 44.650 | 19.040 | 10.790 | 0.050 | 0.930 | 15.020 | 7.150 | 1.180 | 0.000 | 0.240 | 0.960 |
|  | **L4** | 44.680 | 30.730 | 5.510 | 0.000 | 0.120 | 8.890 | 8.470 | 1.370 | 0.000 | 0.130 | 0.100 |
|  | **L5** | 0.000 | 0.000 | 0.000 | 0.000 | 0.000 | 100.000 | 0.000 | 0.000 | 0.000 | 0.000 | 0.000 |
| **6km** | **L1** | 39.030 | 28.630 | 7.690 | 0.310 | 0.530 | 8.470 | 12.200 | 1.420 | 1.150 | 0.380 | 0.180 |
|  | **L2** | 69.910 | 9.340 | 8.170 | 0.190 | 0.500 | 6.040 | 4.210 | 1.180 | 0.000 | 0.110 | 0.360 |
|  | **L3** | 41.890 | 24.060 | 11.250 | 0.420 | 0.850 | 10.510 | 7.400 | 0.830 | 0.910 | 0.290 | 1.590 |
|  | **L4** | 43.250 | 34.730 | 6.110 | 0.000 | 0.160 | 5.400 | 8.700 | 1.160 | 0.000 | 0.200 | 0.200 |
|  | **L5** | 0.010 | 5.490 | 2.070 | 0.040 | 0.140 | 89.130 | 0.530 | 0.010 | 2.520 | 0.030 | 0.040 |
|  |  |  |  |  |  |  |  |  |  |  |  |  |
| **Scale** | **Landscape** | **Qi_agriculture_GLS** | **Qi_pasture_GLS** | **Qi_riparian_forest_GLS** | **Qi_roads_GLS** | **Qi_rural_build_GLS** | **Qi_savana_GLS** | **Qi_seasonal_forest_GLS** | **Qi_tree_plantation_GLS** | **Qi_urban_area_GLS** | **Qi_water_GLS** | **Qi_wet_grassland_GLS** |
| **2km** | **L1** | 0.900 | 0.500 | 0.200 | 0.800 | 0.500 | 0.100 | 0.100 | 0.600 | 0.300 | 0.500 | 0.400 |
|  | **L2** | 0.900 | 0.500 | 0.200 | 0.800 | 0.500 | 0.100 | 0.100 | 0.600 | 0.300 | 0.500 | 0.400 |
|  | **L3** | 0.900 | 0.500 | 0.200 | 0.800 | 0.500 | 0.100 | 0.100 | 0.600 | 0.300 | 0.500 | 0.400 |
|  | **L4** | 0.900 | 0.500 | 0.200 | 0.800 | 0.500 | 0.100 | 0.100 | 0.600 | 0.300 | 0.500 | 0.400 |
|  | **L5** | 0.900 | 0.500 | 0.200 | 0.800 | 0.500 | 0.100 | 0.100 | 0.600 | 0.300 | 0.500 | 0.400 |
| **4km** | **L1** | 0.900 | 0.500 | 0.200 | 0.800 | 0.500 | 0.100 | 0.100 | 0.600 | 0.300 | 0.500 | 0.400 |
|  | **L2** | 0.900 | 0.500 | 0.200 | 0.800 | 0.500 | 0.100 | 0.100 | 0.600 | 0.300 | 0.500 | 0.400 |
|  | **L3** | 0.900 | 0.500 | 0.200 | 0.800 | 0.500 | 0.100 | 0.100 | 0.600 | 0.300 | 0.500 | 0.400 |
|  | **L4** | 0.900 | 0.500 | 0.200 | 0.800 | 0.500 | 0.100 | 0.100 | 0.600 | 0.300 | 0.500 | 0.400 |
|  | **L5** | 0.900 | 0.500 | 0.200 | 0.800 | 0.500 | 0.100 | 0.100 | 0.600 | 0.300 | 0.500 | 0.400 |
| **6km** | **L1** | 0.900 | 0.500 | 0.200 | 0.800 | 0.500 | 0.100 | 0.100 | 0.600 | 0.300 | 0.500 | 0.400 |
|  | **L2** | 0.900 | 0.500 | 0.200 | 0.800 | 0.500 | 0.100 | 0.100 | 0.600 | 0.300 | 0.500 | 0.400 |
|  | **L3** | 0.900 | 0.500 | 0.200 | 0.800 | 0.500 | 0.100 | 0.100 | 0.600 | 0.300 | 0.500 | 0.400 |
|  | **L4** | 0.900 | 0.500 | 0.200 | 0.800 | 0.500 | 0.100 | 0.100 | 0.600 | 0.300 | 0.500 | 0.400 |
|  | **L5** | 0.900 | 0.500 | 0.200 | 0.800 | 0.500 | 0.100 | 0.100 | 0.600 | 0.300 | 0.500 | 0.400 |
|  |  |  |  |  |  |  |  |  |  |  |  |  |
| **Scale** | **Landscape** | **Qi_agriculture_TAT** | **Qi_pasture_TAT** | **Qi_riparian_forest_TAT** | **Qi_roads_TAT** | **Qi_rural_build_TAT** | **Qi_savana_TAT** | **Qi_seasonal_forest_TAT** | **Qi_tree_plantation_TAT** | **Qi_urban_area_TAT** | **Qi_water_TAT** | **Qi_wet_grassland_TAT** |
| **2km** | **L1** | 0.400 | 0.400 | 0.100 | 0.600 | 1.000 | 0.100 | 0.100 | 0.300 | 1.000 | 0.100 | 0.100 |
|  | **L2** | 0.400 | 0.400 | 0.100 | 0.600 | 1.000 | 0.100 | 0.100 | 0.300 | 1.000 | 0.100 | 0.100 |
|  | **L3** | 0.400 | 0.400 | 0.100 | 0.600 | 1.000 | 0.100 | 0.100 | 0.300 | 1.000 | 0.100 | 0.100 |
|  | **L4** | 0.400 | 0.400 | 0.100 | 0.600 | 1.000 | 0.100 | 0.100 | 0.300 | 1.000 | 0.100 | 0.100 |
|  | **L5** | 0.400 | 0.400 | 0.100 | 0.600 | 1.000 | 0.100 | 0.100 | 0.300 | 1.000 | 0.100 | 0.100 |
| **4km** | **L1** | 0.400 | 0.400 | 0.100 | 0.600 | 1.000 | 0.100 | 0.100 | 0.300 | 1.000 | 0.100 | 0.100 |
|  | **L2** | 0.400 | 0.400 | 0.100 | 0.600 | 1.000 | 0.100 | 0.100 | 0.300 | 1.000 | 0.100 | 0.100 |
|  | **L3** | 0.400 | 0.400 | 0.100 | 0.600 | 1.000 | 0.100 | 0.100 | 0.300 | 1.000 | 0.100 | 0.100 |
|  | **L4** | 0.400 | 0.400 | 0.100 | 0.600 | 1.000 | 0.100 | 0.100 | 0.300 | 1.000 | 0.100 | 0.100 |
|  | **L5** | 0.400 | 0.400 | 0.100 | 0.600 | 1.000 | 0.100 | 0.100 | 0.300 | 1.000 | 0.100 | 0.100 |
| **6km** | **L1** | 0.400 | 0.400 | 0.100 | 0.600 | 1.000 | 0.100 | 0.100 | 0.300 | 1.000 | 0.100 | 0.100 |
|  | **L2** | 0.400 | 0.400 | 0.100 | 0.600 | 1.000 | 0.100 | 0.100 | 0.300 | 1.000 | 0.100 | 0.100 |
|  | **L3** | 0.400 | 0.400 | 0.100 | 0.600 | 1.000 | 0.100 | 0.100 | 0.300 | 1.000 | 0.100 | 0.100 |
|  | **L4** | 0.400 | 0.400 | 0.100 | 0.600 | 1.000 | 0.100 | 0.100 | 0.300 | 1.000 | 0.100 | 0.100 |
|  | **L5** | 0.400 | 0.400 | 0.100 | 0.600 | 1.000 | 0.100 | 0.100 | 0.300 | 1.000 | 0.100 | 0.100 |
|  |  |  |  |  |  |  |  |  |  |  |  |  |
| **Scale** | **Landscape** | **Qi_agriculture_MA** | **Qi_pasture_MA** | **Qi_riparian_forest_MA** | **Qi_roads_MA** | **Qi_rural_build_MA** | **Qi_savana_MA** | **Qi_seasonal_forest_MA** | **Qi_tree_plantation_MA** | **Qi_urban_area_MA** | **Qi_water_MA** | **Qi_wet_grassland_MA** |
| **2km** | **L1** | 0.100 | 0.100 | 0.200 | 1.000 | 1.000 | 0.100 | 0.100 | 0.100 | 1.000 | 0.500 | 0.400 |
|  | **L2** | 0.100 | 0.100 | 0.200 | 1.000 | 1.000 | 0.100 | 0.100 | 0.100 | 1.000 | 0.500 | 0.400 |
|  | **L3** | 0.100 | 0.100 | 0.200 | 1.000 | 1.000 | 0.100 | 0.100 | 0.100 | 1.000 | 0.500 | 0.400 |
|  | **L4** | 0.100 | 0.100 | 0.200 | 1.000 | 1.000 | 0.100 | 0.100 | 0.100 | 1.000 | 0.500 | 0.400 |
|  | **L5** | 0.100 | 0.100 | 0.200 | 1.000 | 1.000 | 0.100 | 0.100 | 0.100 | 1.000 | 0.500 | 0.400 |
| **4km** | **L1** | 0.100 | 0.100 | 0.200 | 1.000 | 1.000 | 0.100 | 0.100 | 0.100 | 1.000 | 0.500 | 0.400 |
|  | **L2** | 0.100 | 0.100 | 0.200 | 1.000 | 1.000 | 0.100 | 0.100 | 0.100 | 1.000 | 0.500 | 0.400 |
|  | **L3** | 0.100 | 0.100 | 0.200 | 1.000 | 1.000 | 0.100 | 0.100 | 0.100 | 1.000 | 0.500 | 0.400 |
|  | **L4** | 0.100 | 0.100 | 0.200 | 1.000 | 1.000 | 0.100 | 0.100 | 0.100 | 1.000 | 0.500 | 0.400 |
|  | **L5** | 0.100 | 0.100 | 0.200 | 1.000 | 1.000 | 0.100 | 0.100 | 0.100 | 1.000 | 0.500 | 0.400 |
| **6km** | **L1** | 0.100 | 0.100 | 0.200 | 1.000 | 1.000 | 0.100 | 0.100 | 0.100 | 1.000 | 0.500 | 0.400 |
|  | **L2** | 0.100 | 0.100 | 0.200 | 1.000 | 1.000 | 0.100 | 0.100 | 0.100 | 1.000 | 0.500 | 0.400 |
|  | **L3** | 0.100 | 0.100 | 0.200 | 1.000 | 1.000 | 0.100 | 0.100 | 0.100 | 1.000 | 0.500 | 0.400 |
|  | **L4** | 0.100 | 0.100 | 0.200 | 1.000 | 1.000 | 0.100 | 0.100 | 0.100 | 1.000 | 0.500 | 0.400 |
|  | **L5** | 0.100 | 0.100 | 0.200 | 1.000 | 1.000 | 0.100 | 0.100 | 0.100 | 1.000 | 0.500 | 0.400 |
|  |  |  |  |  |  |  |  |  |  |  |  |  |
| **Scale** | **Landscape** | **Qi_agriculture_DA** | **Qi_pasture_DA** | **Qi_riparian_forest_DA** | **Qi_roads_DA** | **Qi_rural_build_DA** | **Qi_savana_DA** | **Qi_seasonal_forest_DA** | **Qi_tree_plantation_DA** | **Qi_urban_area_DA** | **Qi_water_DA** | **Qi_wet_grassland_DA** |
| **2km** | **L1** | 0.400 | 0.400 | 0.100 | 0.100 | 1.000 | 0.100 | 0.100 | 0.200 | 1.000 | 1.000 | 0.500 |
|  | **L2** | 0.400 | 0.400 | 0.100 | 0.100 | 1.000 | 0.100 | 0.100 | 0.200 | 1.000 | 1.000 | 0.500 |
|  | **L3** | 0.400 | 0.400 | 0.100 | 0.100 | 1.000 | 0.100 | 0.100 | 0.200 | 1.000 | 1.000 | 0.500 |
|  | **L4** | 0.400 | 0.400 | 0.100 | 0.100 | 1.000 | 0.100 | 0.100 | 0.200 | 1.000 | 1.000 | 0.500 |
|  | **L5** | 0.400 | 0.400 | 0.100 | 0.100 | 1.000 | 0.100 | 0.100 | 0.200 | 1.000 | 1.000 | 0.500 |
| **4km** | **L1** | 0.400 | 0.400 | 0.100 | 0.100 | 1.000 | 0.100 | 0.100 | 0.200 | 1.000 | 1.000 | 0.500 |
|  | **L2** | 0.400 | 0.400 | 0.100 | 0.100 | 1.000 | 0.100 | 0.100 | 0.200 | 1.000 | 1.000 | 0.500 |
|  | **L3** | 0.400 | 0.400 | 0.100 | 0.100 | 1.000 | 0.100 | 0.100 | 0.200 | 1.000 | 1.000 | 0.500 |
|  | **L4** | 0.400 | 0.400 | 0.100 | 0.100 | 1.000 | 0.100 | 0.100 | 0.200 | 1.000 | 1.000 | 0.500 |
|  | **L5** | 0.400 | 0.400 | 0.100 | 0.100 | 1.000 | 0.100 | 0.100 | 0.200 | 1.000 | 1.000 | 0.500 |
| **6km** | **L1** | 0.400 | 0.400 | 0.100 | 0.100 | 1.000 | 0.100 | 0.100 | 0.200 | 1.000 | 1.000 | 0.500 |
|  | **L2** | 0.400 | 0.400 | 0.100 | 0.100 | 1.000 | 0.100 | 0.100 | 0.200 | 1.000 | 1.000 | 0.500 |
|  | **L3** | 0.400 | 0.400 | 0.100 | 0.100 | 1.000 | 0.100 | 0.100 | 0.200 | 1.000 | 1.000 | 0.500 |
|  | **L4** | 0.400 | 0.400 | 0.100 | 0.100 | 1.000 | 0.100 | 0.100 | 0.200 | 1.000 | 1.000 | 0.500 |
|  | **L5** | 0.400 | 0.400 | 0.100 | 0.100 | 1.000 | 0.100 | 0.100 | 0.200 | 1.000 | 1.000 | 0.500 |
|  |  |  |  |  |  |  |  |  |  |  |  |  |
| **Scale** | **Landscape** | **Qi_agriculture_CHB** | **Qi_pasture_CHB** | **Qi_riparian_forest_CHB** | **Qi_roads_CHB** | **Qi_rural_build_CHB** | **Qi_savana_CHB** | **Qi_seasonal_forest_CHB** | **Qi_tree_plantation_CHB** | **Qi_urban_area_CHB** | **Qi_water_CHB** | **Qi_wet_grassland_CHB** |
| **2km** | **L1** | 0.100 | 0.100 | 0.900 | 0.100 | 1.000 | 0.100 | 0.900 | 0.200 | 0.800 | 1.000 | 0.800 |
|  | **L2** | 0.100 | 0.100 | 0.900 | 0.100 | 1.000 | 0.100 | 0.900 | 0.200 | 0.800 | 1.000 | 0.800 |
|  | **L3** | 0.100 | 0.100 | 0.900 | 0.100 | 1.000 | 0.100 | 0.900 | 0.200 | 0.800 | 1.000 | 0.800 |
|  | **L4** | 0.100 | 0.100 | 0.900 | 0.100 | 1.000 | 0.100 | 0.900 | 0.200 | 0.800 | 1.000 | 0.800 |
|  | **L5** | 0.100 | 0.100 | 0.900 | 0.100 | 1.000 | 0.100 | 0.900 | 0.200 | 0.800 | 1.000 | 0.800 |
| **4km** | **L1** | 0.100 | 0.100 | 0.900 | 0.100 | 1.000 | 0.100 | 0.900 | 0.200 | 0.800 | 1.000 | 0.800 |
|  | **L2** | 0.100 | 0.100 | 0.900 | 0.100 | 1.000 | 0.100 | 0.900 | 0.200 | 0.800 | 1.000 | 0.800 |
|  | **L3** | 0.100 | 0.100 | 0.900 | 0.100 | 1.000 | 0.100 | 0.900 | 0.200 | 0.800 | 1.000 | 0.800 |
|  | **L4** | 0.100 | 0.100 | 0.900 | 0.100 | 1.000 | 0.100 | 0.900 | 0.200 | 0.800 | 1.000 | 0.800 |
|  | **L5** | 0.100 | 0.100 | 0.900 | 0.100 | 1.000 | 0.100 | 0.900 | 0.200 | 0.800 | 1.000 | 0.800 |
| **6km** | **L1** | 0.100 | 0.100 | 0.900 | 0.100 | 1.000 | 0.100 | 0.900 | 0.200 | 0.800 | 1.000 | 0.800 |
|  | **L2** | 0.100 | 0.100 | 0.900 | 0.100 | 1.000 | 0.100 | 0.900 | 0.200 | 0.800 | 1.000 | 0.800 |
|  | **L3** | 0.100 | 0.100 | 0.900 | 0.100 | 1.000 | 0.100 | 0.900 | 0.200 | 0.800 | 1.000 | 0.800 |
|  | **L4** | 0.100 | 0.100 | 0.900 | 0.100 | 1.000 | 0.100 | 0.900 | 0.200 | 0.800 | 1.000 | 0.800 |
|  | **L5** | 0.100 | 0.100 | 0.900 | 0.100 | 1.000 | 0.100 | 0.900 | 0.200 | 0.800 | 1.000 | 0.800 |
|  |  |  |  |  |  |  |  |  |  |  |  |  |
| **Scale** | **Landscape** | **Qi_agriculture_pca** | **Qi_pasture_pca** | **Qi_riparian_pca** | **Qi_roads_pca** | **Qi_rural_pca** | **Qi_savana_pca** | **Qi_seasonal_pca** | **Qi_tree_pca** | **Qi_urban_pca** | **Qi_water_pca** | **Qi_wet_pca** |
| **2km** | **L1** | 0.274 | 0.227 | 0.267 | 0.328 | 1.000 | 0.100 | 0.233 | 0.254 | 0.955 | 0.625 | 0.426 |
|  | **L2** | 0.274 | 0.227 | 0.267 | 0.328 | 1.000 | 0.100 | 0.233 | 0.254 | 0.955 | 0.625 | 0.426 |
|  | **L3** | 0.274 | 0.227 | 0.267 | 0.328 | 1.000 | 0.100 | 0.233 | 0.254 | 0.955 | 0.625 | 0.426 |
|  | **L4** | 0.274 | 0.227 | 0.267 | 0.328 | 1.000 | 0.100 | 0.233 | 0.254 | 0.955 | 0.625 | 0.426 |
|  | **L5** | 0.274 | 0.227 | 0.267 | 0.328 | 1.000 | 0.100 | 0.233 | 0.254 | 0.955 | 0.625 | 0.426 |
| **4km** | **L1** | 0.274 | 0.227 | 0.267 | 0.328 | 1.000 | 0.100 | 0.233 | 0.254 | 0.955 | 0.625 | 0.426 |
|  | **L2** | 0.274 | 0.227 | 0.267 | 0.328 | 1.000 | 0.100 | 0.233 | 0.254 | 0.955 | 0.625 | 0.426 |
|  | **L3** | 0.274 | 0.227 | 0.267 | 0.328 | 1.000 | 0.100 | 0.233 | 0.254 | 0.955 | 0.625 | 0.426 |
|  | **L4** | 0.274 | 0.227 | 0.267 | 0.328 | 1.000 | 0.100 | 0.233 | 0.254 | 0.955 | 0.625 | 0.426 |
|  | **L5** | 0.274 | 0.227 | 0.267 | 0.328 | 1.000 | 0.100 | 0.233 | 0.254 | 0.955 | 0.625 | 0.426 |
| **6km** | **L1** | 0.274 | 0.227 | 0.267 | 0.328 | 1.000 | 0.100 | 0.233 | 0.254 | 0.955 | 0.625 | 0.426 |
|  | **L2** | 0.274 | 0.227 | 0.267 | 0.328 | 1.000 | 0.100 | 0.233 | 0.254 | 0.955 | 0.625 | 0.426 |
|  | **L3** | 0.274 | 0.227 | 0.267 | 0.328 | 1.000 | 0.100 | 0.233 | 0.254 | 0.955 | 0.625 | 0.426 |
|  | **L4** | 0.274 | 0.227 | 0.267 | 0.328 | 1.000 | 0.100 | 0.233 | 0.254 | 0.955 | 0.625 | 0.426 |
|  | **L5** | 0.274 | 0.227 | 0.267 | 0.328 | 1.000 | 0.100 | 0.233 | 0.254 | 0.955 | 0.625 | 0.426 |

**Table S6**. Landscape quality (LQ) of *Caryocar brasiliense* pollinator and dispersers based on buffers of 0.5 km drew around sampling sites. GLS, *Glossophaga soricina*; TAT, *Tapirus terrestris*; MA, *Mazama americana* and *M. guazoupira*; DA, *Dasyprocta* sp.; CHB, *Chrysocyon brachyurus*; PCA, PCA resistance value calculated for the pollinator and dispersers of *C. brasiliense*.

| **Site** | **LQ_GLS** | **LQ_TAT** | **LQ_DA** | **LQ_CHB** | **LQ_MA** | **LQ_PCA** |
| --- | --- | --- | --- | --- | --- | --- |
| **L1F1** | 0.572 | 0.742 | 0.742 | 0.784 | 0.881 | 0.792 |
| **L1F2** | 0.315 | 0.663 | 0.691 | 0.855 | 0.881 | 0.756 |
| **L2F3** | 0.235 | 0.651 | 0.651 | 0.894 | 0.899 | 0.754 |
| **L2F4** | 0.396 | 0.705 | 0.709 | 0.855 | 0.885 | 0.776 |
| **L3F5** | 0.662 | 0.791 | 0.791 | 0.680 | 0.871 | 0.796 |
| **L3F6** | 0.662 | 0.791 | 0.791 | 0.680 | 0.871 | 0.796 |
| **L4F7** | 0.334 | 0.682 | 0.688 | 0.806 | 0.890 | 0.755 |
| **L4F8** | 0.414 | 0.687 | 0.691 | 0.814 | 0.891 | 0.768 |
| **L5F9** | 0.829 | 0.825 | 0.825 | 0.833 | 0.863 | 0.843 |
| **L5F10** | 0.829 | 0.825 | 0.825 | 0.833 | 0.863 | 0.843 |

**Table S7**. Landscape quality (LQ) of *Caryocar brasiliense* pollinator and dispersers based on buffers of 2km, 4km and 6km for five landscapes. GLS, *Glossophaga soricina*; TAT, *Tapirus terrestris*; MA*, Mazama americana* and *M. guazoupira*; DA, *Dasyprocta* sp.; CHB, *Chrysocyon brachyurus*; PCA, PCA resistance value calculated for the pollinator and dispersers of *C. brasiliense*.

| **Landscape** | **Scale** | **LQ_GLS** | **LQ_TAT** | **LQ_DA** | **LQ_CHB** | **LQ_MA** | **LQ_PCA** |
| --- | --- | --- | --- | --- | --- | --- | --- |
| **L1** | **2km** | 0.512 | 0.731 | 0.731 | 0.707 | 0.888 | 0.787 |
| **L2** | **2km** | 0.325 | 0.678 | 0.681 | 0.815 | 0.890 | 0.765 |
| **L3** | **2km** | 0.628 | 0.770 | 0.768 | 0.597 | 0.870 | 0.788 |
| **L4** | **2km** | 0.408 | 0.685 | 0.686 | 0.750 | 0.892 | 0.765 |
| **L5** | **2km** | 0.900 | 0.900 | 0.900 | 0.700 | 0.900 | 0.900 |
| **L1** | **4km** | 0.432 | 0.673 | 0.673 | 0.692 | 0.865 | 0.743 |
| **L2** | **4km** | 0.289 | 0.657 | 0.656 | 0.777 | 0.887 | 0.744 |
| **L3** | **4km** | 0.442 | 0.698 | 0.693 | 0.689 | 0.877 | 0.756 |
| **L4** | **4km** | 0.406 | 0.670 | 0.670 | 0.749 | 0.893 | 0.759 |
| **L5** | **4km** | 0.900 | 0.900 | 0.900 | 0.700 | 0.900 | 0.900 |
| **L1** | **6km** | 0.450 | 0.677 | 0.676 | 0.680 | 0.872 | 0.747 |
| **L2** | **6km** | 0.285 | 0.654 | 0.654 | 0.765 | 0.884 | 0.739 |
| **L3** | **6km** | 0.439 | 0.683 | 0.677 | 0.681 | 0.863 | 0.744 |
| **L4** | **6km** | 0.401 | 0.662 | 0.661 | 0.748 | 0.891 | 0.754 |
| **L5** | **6km** | 0.870 | 0.859 | 0.859 | 0.674 | 0.873 | 0.866 |

**Table S8**. Sampling size for neutral and quantitative genetic analysis of *Caryocar brasiliense* in 10 sites of savanna in the Cerrado biome, Brazil. For neutral genetic analysis, we collected expanded leaves of adults and juveniles. For quantitative genetic data, the number of mother-trees corresponds to the number of trees in which fruits were collected for the experiment in nursery. The number of fruits are the total number of fruits collected in each site. The number of seeds are the total number of seeds planted at the nursery and the number of seedlings are the total seedlings grown in nursery in each site.

| **Site** | **Neutral genetic data** | | **Quantitative genetic data** | | | |
| --- | --- | --- | --- | --- | --- | --- |
|  | **Number of adults** | **Number of juveniles** | **Number of mother-trees** | **Number of fruits** | **Number of seeds** | **Number of seedlings** |
| L1F1 | 70 | 55 | 10 | 151 | 250 | 43 |
| L1F2 | 65 | 56 | 8 | 130 | 215 | 33 |
| L2F3 | 70 | 54 | 10 | 171 | 240 | 13 |
| L2F4 | 66 | 43 | 10 | 163 | 246 | 9 |
| L3F5 | 70 | 54 | 10 | 202 | 340 | 15 |
| L3F6 | 63 | 35 | 2 | 44 | 66 | 6 |
| L4F7 | 70 | 27 | 10 | 177 | 299 | 35 |
| L4F8 | 76 | 39 | 10 | 164 | 285 | 45 |
| L5F9 | 61 | 28 | 10 | 182 | 314 | 25 |
| L5F10 | 66 | 31 | 10 | 186 | 306 | 26 |
| Total | 677 | 422 | 90 | 1570 | 2561 | 250 |

**Table S9**. Average of adaptive quantitative traits of *Caryocar brasiliense* measured in seeds and seedlings from 10 sites in the Cerrado biome, Brazil. SLD, seed longitudinal diameter (mm); STD, seed transversal diameter (mm); SM, seed mass (g); PG, proportion of germinated seeds; TG, time to germination (days); IH, initial height (cm); FH, final height (cm); HGR, height growth rate (cm/day); ID, initial diameter (mm); FD, final diameter (mm); DGR, diameter growth rate (mm/day); NL, number of leaves; LL, leaf length (mm); LW, leaf width (mm); RL, root length (cm); RGM, root green mass (g); RDM, root dry mass (g); ASL, aboveground shoot length (cm); AGM, aboveground green mass (g); ADM, aboveground dry mass (g).

| **Site** | **SLD** | **STD** | **SM** | **PG** | **TG** | **IH** | **FH** | **HGR** | **ID** | **FD** | **DGR** | **NL** | **LL** |
| --- | --- | --- | --- | --- | --- | --- | --- | --- | --- | --- | --- | --- | --- |
| **L1F1** | 34.833 | 21.756 | 9.563 | 18.777 | 48.341 | 6.900 | 16.703 | 2.680 | 3.810 | 5.719 | 0.476 | 4.231 | 77.068 |
| **L1F2** | 32.501 | 20.465 | 7.495 | 16.418 | 42.219 | 8.075 | 16.597 | 2.413 | 3.717 | 5.385 | 0.473 | 4.031 | 75.241 |
| **L2F3** | 33.067 | 21.180 | 8.373 | 5.532 | 37.308 | 7.777 | 15.185 | 2.101 | 3.841 | 5.839 | 0.539 | 4.917 | 74.769 |
| **L2F4** | 33.222 | 20.398 | 7.817 | 4.018 | 44.222 | 7.213 | 15.663 | 2.037 | 4.523 | 6.143 | 0.446 | 5.000 | 72.504 |
| **L3F5** | 31.172 | 20.227 | 6.826 | 5.172 | 40.571 | 8.415 | 17.877 | 2.466 | 4.058 | 6.063 | 0.582 | 4.846 | 80.681 |
| **L3F6** | 30.239 | 19.847 | 7.311 | 12.000 | 39.167 | 7.650 | 16.917 | 2.521 | 4.072 | 5.785 | 0.519 | 5.500 | 81.464 |
| **L4F7** | 32.914 | 20.377 | 7.108 | 13.780 | 49.441 | 6.661 | 15.656 | 2.424 | 3.659 | 5.253 | 0.450 | 3.781 | 73.252 |
| **L4F8** | 33.377 | 20.393 | 7.467 | 18.672 | 46.512 | 6.874 | 15.288 | 2.382 | 4.046 | 6.064 | 0.573 | 4.317 | 77.644 |
| **L5F9** | 32.473 | 20.368 | 6.759 | 8.711 | 47.167 | 7.725 | 18.246 | 3.052 | 4.248 | 6.253 | 0.559 | 4.292 | 79.032 |
| **L5F10** | 32.297 | 20.395 | 6.124 | 9.059 | 43.038 | 7.081 | 14.112 | 1.981 | 3.697 | 5.277 | 0.418 | 4.458 | 66.818 |
|  |  |  |  |  |  |  |  |  |  |  |  |  |  |
| **Site** | **LW** | **RL** | **RGM** | **RDM** | **ASL** | **AGM** | **ADM** |  |  |  |  |  |  |
| **L1F1** | 48.020 | 25.741 | 8.565 | 3.444 | 18.885 | 7.934 | 2.589 |  |  |  |  |  |  |
| **L1F2** | 47.918 | 23.634 | 8.184 | 3.085 | 18.316 | 6.676 | 2.348 |  |  |  |  |  |  |
| **L2F3** | 47.730 | 24.942 | 9.538 | 3.767 | 17.783 | 7.838 | 2.639 |  |  |  |  |  |  |
| **L2F4** | 48.254 | 23.963 | 9.584 | 3.838 | 17.113 | 8.208 | 3.071 |  |  |  |  |  |  |
| **L3F5** | 49.764 | 26.723 | 9.422 | 3.638 | 19.838 | 8.598 | 3.072 |  |  |  |  |  |  |
| **L3F6** | 46.250 | 24.650 | 7.428 | 2.897 | 17.133 | 7.246 | 2.667 |  |  |  |  |  |  |
| **L4F7** | 46.265 | 23.639 | 8.079 | 3.168 | 17.948 | 7.397 | 2.514 |  |  |  |  |  |  |
| **L4F8** | 48.383 | 24.298 | 8.623 | 3.355 | 16.808 | 7.550 | 2.606 |  |  |  |  |  |  |
| **L5F9** | 48.599 | 25.102 | 9.320 | 3.513 | 20.522 | 9.141 | 3.158 |  |  |  |  |  |  |
| **L5F10** | 42.964 | 24.263 | 8.806 | 3.309 | 16.551 | 6.609 | 2.277 |  |  |  |  |  |  |

**Table S10**. Correlation among adaptive quantitative traits measured in seeds and seedlings of *Caryocar brasiliense* from 10 sites in the Cerrado biome, Brazil. SLD, seed longitudinal diameter (mm); STD, seed transversal diameter (mm); SM, seed mass (g); PG, proportion of germinated seeds; TG, time to germination (days); IH, initial height (cm); FH, final height (cm); HGR, height growth rate (cm/day); ID, initial diameter (mm); FD, final diameter (mm); DGR, diameter growth rate (mm/day); NL, number of leaves; LL, leaf length (mm); LW, leaf width (mm); RL, root length (cm); RGM, root green mass (g); RDM, root dry mass (g); ASL, aboveground shoot length (cm); AGM, aboveground green mass (g); ADM, aboveground green mass (g).

|  | **SLD** | **STD** | **SM** | **TG** | **IH** | **FH** | **HGR** | **ID** | **FD** | **DGR** | **NL** | **LL** | **LW** | **RL** | **RDM** | **RGM** | **ASL** | **ADM** | **AGM** | **PG** |
| --- | --- | --- | --- | --- | --- | --- | --- | --- | --- | --- | --- | --- | --- | --- | --- | --- | --- | --- | --- | --- |
| **SLD** | 1 | 0.893 | -0.092 | -0.425 | 0.456 | -0.180 | -0.378 | -0.180 | -0.608 | -0.768 | -0.383 | 0.265 | -0.106 | 0.316 | -0.164 | -0.161 | -0.107 | 0.055 | -0.039 | -0.183 |
| **STD** | 0.893 | 1 | -0.145 | -0.572 | 0.343 | -0.083 | -0.272 | -0.117 | -0.558 | -0.875 | -0.103 | 0.407 | 0.052 | 0.580 | 0.037 | 0.112 | 0.032 | 0.201 | 0.163 | -0.099 |
| **SM** | -0.092 | -0.145 | 1 | -0.369 | -0.555 | -0.170 | 0.721 | 0.875 | -0.334 | -0.020 | 0.305 | -0.411 | 0.313 | -0.639 | -0.443 | -0.687 | 0.334 | 0.470 | 0.021 | -0.546 |
| **TG** | -0.425 | -0.572 | -0.369 | 1 | 0.058 | 0.316 | 0.035 | -0.184 | 0.159 | 0.747 | -0.199 | 0.040 | -0.037 | -0.140 | 0.135 | 0.210 | 0.125 | -0.212 | 0.084 | 0.530 |
| **IH** | 0.456 | 0.343 | -0.555 | 0.058 | 1 | 0.240 | -0.699 | -0.447 | 0.161 | -0.288 | -0.312 | 0.110 | -0.643 | 0.137 | 0.041 | -0.030 | -0.390 | -0.377 | -0.307 | -0.236 |
| **FH** | -0.180 | -0.083 | -0.170 | 0.316 | 0.240 | 1 | 0.298 | 0.133 | 0.402 | 0.305 | 0.683 | 0.574 | 0.390 | 0.181 | 0.434 | 0.123 | 0.668 | 0.592 | 0.699 | -0.133 |
| **HGR** | -0.378 | -0.272 | 0.721 | 0.035 | -0.699 | 0.298 | 1 | 0.863 | -0.146 | 0.363 | 0.666 | -0.009 | 0.660 | -0.260 | 0.112 | -0.147 | 0.762 | 0.756 | 0.575 | -0.109 |
| **ID** | -0.180 | -0.117 | 0.875 | -0.184 | -0.447 | 0.133 | 0.863 | 1 | -0.306 | 0.084 | 0.520 | -0.295 | 0.355 | -0.467 | -0.160 | -0.441 | 0.556 | 0.606 | 0.255 | -0.449 |
| **FD** | -0.608 | -0.558 | -0.334 | 0.159 | 0.161 | 0.402 | -0.146 | -0.306 | 1 | 0.521 | 0.359 | 0.055 | -0.181 | 0.002 | 0.444 | 0.242 | -0.161 | -0.144 | 0.004 | -0.058 |
| **DGR** | -0.768 | -0.875 | -0.020 | 0.747 | -0.288 | 0.305 | 0.363 | 0.084 | 0.521 | 1 | 0.180 | -0.180 | 0.099 | -0.298 | 0.332 | 0.150 | 0.194 | -0.013 | 0.160 | 0.272 |
| **NL** | -0.383 | -0.103 | 0.305 | -0.199 | -0.312 | 0.683 | 0.666 | 0.520 | 0.359 | 0.180 | 1 | 0.301 | 0.628 | 0.150 | 0.439 | 0.116 | 0.737 | 0.798 | 0.733 | -0.302 |
| **LL** | 0.265 | 0.407 | -0.411 | 0.040 | 0.110 | 0.574 | -0.009 | -0.295 | 0.055 | -0.180 | 0.301 | 1 | 0.434 | 0.585 | 0.339 | 0.379 | 0.404 | 0.445 | 0.639 | 0.351 |
| **LW** | -0.106 | 0.052 | 0.313 | -0.037 | -0.643 | 0.390 | 0.660 | 0.355 | -0.181 | 0.099 | 0.628 | 0.434 | 1 | 0.257 | 0.181 | 0.089 | 0.877 | 0.841 | 0.859 | 0.054 |
| **RL** | 0.316 | 0.580 | -0.639 | -0.140 | 0.137 | 0.181 | -0.260 | -0.467 | 0.002 | -0.298 | 0.150 | 0.585 | 0.257 | 1 | 0.684 | 0.822 | 0.149 | 0.147 | 0.497 | 0.422 |
| **DRM** | -0.164 | 0.037 | -0.443 | 0.135 | 0.041 | 0.434 | 0.112 | -0.160 | 0.444 | 0.332 | 0.439 | 0.339 | 0.181 | 0.684 | 1 | 0.800 | 0.257 | 0.239 | 0.559 | 0.308 |
| **GRM** | -0.161 | 0.112 | -0.687 | 0.210 | -0.030 | 0.123 | -0.147 | -0.441 | 0.242 | 0.150 | 0.116 | 0.379 | 0.089 | 0.822 | 0.800 | 1 | 0.016 | -0.092 | 0.352 | 0.710 |
| **ASL** | -0.107 | 0.032 | 0.334 | 0.125 | -0.390 | 0.668 | 0.762 | 0.556 | -0.161 | 0.194 | 0.737 | 0.404 | 0.877 | 0.149 | 0.257 | 0.016 | 1 | 0.921 | 0.907 | -0.094 |
| **DSM** | 0.055 | 0.201 | 0.470 | -0.212 | -0.377 | 0.592 | 0.756 | 0.606 | -0.144 | -0.013 | 0.798 | 0.445 | 0.841 | 0.147 | 0.239 | -0.092 | 0.921 | 1 | 0.864 | -0.282 |
| **GSM** | -0.039 | 0.163 | 0.021 | 0.084 | -0.307 | 0.699 | 0.575 | 0.255 | 0.004 | 0.160 | 0.733 | 0.639 | 0.859 | 0.497 | 0.559 | 0.352 | 0.907 | 0.864 | 1 | 0.112 |
| **PG** | -0.183 | -0.099 | -0.546 | 0.530 | -0.236 | -0.133 | -0.109 | -0.449 | -0.058 | 0.272 | -0.302 | 0.351 | 0.054 | 0.422 | 0.308 | 0.710 | -0.094 | -0.282 | 0.112 | 1 |

**Table S11**. Correlation among the average of adaptive quantitative traits measured in seeds and seedling of *Caryocar brasiliense* from five landscapes in the Cerrado biome, Brazil. SLD, seed longitudinal diameter (mm); STD, seed transversal diameter (mm); SM, seed mass (g); PG, proportion of germinated seeds; TG, time to germination (days); IH, initial height (cm); FH, final height (cm); HGR, height growth rate (cm/day); ID, initial diameter (mm); FD, final diameter (mm); DGR, diameter growth rate (mm/day); NL, number of leaves; LL, leaf length (mm); LW, leaf width (mm); RL, root length (cm); RGM, root green mass (g); RDM, root dry mass (g); ASL, aboveground shoot length (cm); AGM, aboveground green mass (g); ADM, aboveground dry mass (g).

|  | **SLD** | **STD** | **SM** | **TG** | **IH** | **FH** | **HGR** | **ID** | **FD** | **DGR** | **NL** | **LL** | **LW** | **RL** | **RDM** | **RGM** | **ASL** | **ADM** | **AGM** | **PG** |
| --- | --- | --- | --- | --- | --- | --- | --- | --- | --- | --- | --- | --- | --- | --- | --- | --- | --- | --- | --- | --- |
| **SLD** | 1 | 0.902 | -0.685 | 0.005 | 0.696 | 0.775 | 0.074 | -0.393 | -0.348 | -0.581 | 0.046 | 0.979 | 0.861 | 0.589 | 0.479 | 0.253 | 0.922 | 0.665 | 0.930 | 0.178 |
| **STD** | 0.902 | 1 | -0.605 | -0.298 | 0.448 | 0.569 | 0.245 | -0.287 | -0.521 | -0.848 | 0.222 | 0.914 | 0.995 | 0.816 | 0.756 | 0.507 | 0.851 | 0.691 | 0.976 | 0.277 |
| **SM** | -0.685 | -0.605 | 1 | -0.542 | -0.431 | -0.204 | 0.554 | 0.926 | 0.810 | 0.196 | 0.563 | -0.558 | -0.541 | -0.717 | -0.365 | -0.645 | -0.410 | 0.064 | -0.493 | -0.554 |
| **TG** | 0.005 | -0.298 | -0.542 | 1 | 0.213 | -0.128 | -0.849 | -0.755 | -0.239 | 0.708 | -0.882 | -0.148 | -0.382 | -0.138 | -0.445 | 0.039 | -0.254 | -0.652 | -0.350 | 0.275 |
| **IH** | 0.696 | 0.448 | -0.431 | 0.213 | 1 | 0.880 | -0.288 | -0.188 | 0.038 | -0.223 | 0.169 | 0.598 | 0.383 | 0.143 | -0.246 | -0.292 | 0.784 | 0.503 | 0.533 | -0.459 |
| **FH** | 0.775 | 0.569 | -0.204 | -0.128 | 0.880 | 1 | 0.186 | 0.105 | 0.255 | -0.398 | 0.411 | 0.773 | 0.532 | 0.070 | -0.031 | -0.387 | 0.914 | 0.816 | 0.712 | -0.448 |
| **HGR** | 0.074 | 0.245 | 0.554 | -0.849 | -0.288 | 0.186 | 1 | 0.709 | 0.421 | -0.474 | 0.654 | 0.272 | 0.316 | -0.090 | 0.458 | -0.164 | 0.238 | 0.661 | 0.347 | -0.075 |
| **ID** | -0.393 | -0.287 | 0.926 | -0.755 | -0.188 | 0.105 | 0.709 | 1 | 0.786 | -0.137 | 0.822 | -0.253 | -0.221 | -0.528 | -0.206 | -0.637 | -0.052 | 0.420 | -0.150 | -0.669 |
| **FD** | -0.348 | -0.521 | 0.810 | -0.239 | 0.038 | 0.255 | 0.421 | 0.786 | 1 | 0.358 | 0.438 | -0.255 | -0.506 | -0.889 | -0.605 | -0.948 | -0.092 | 0.239 | -0.328 | -0.699 |
| **DGR** | -0.581 | -0.848 | 0.196 | 0.708 | -0.223 | -0.398 | -0.474 | -0.137 | 0.358 | 1 | -0.622 | -0.638 | -0.888 | -0.740 | -0.734 | -0.418 | -0.691 | -0.743 | -0.827 | -0.016 |
| **NL** | 0.046 | 0.222 | 0.563 | -0.882 | 0.169 | 0.411 | 0.654 | 0.822 | 0.438 | -0.622 | 1 | 0.148 | 0.281 | -0.029 | 0.079 | -0.364 | 0.405 | 0.736 | 0.327 | -0.674 |
| **LL** | 0.979 | 0.914 | -0.558 | -0.148 | 0.598 | 0.773 | 0.272 | -0.253 | -0.255 | -0.638 | 0.148 | 1 | 0.888 | 0.544 | 0.561 | 0.219 | 0.925 | 0.760 | 0.960 | 0.183 |
| **LW** | 0.861 | 0.995 | -0.541 | -0.382 | 0.383 | 0.532 | 0.316 | -0.221 | -0.506 | -0.888 | 0.281 | 0.888 | 1 | 0.819 | 0.795 | 0.518 | 0.827 | 0.710 | 0.970 | 0.274 |
| **RL** | 0.589 | 0.816 | -0.717 | -0.138 | 0.143 | 0.070 | -0.090 | -0.528 | -0.889 | -0.740 | -0.029 | 0.544 | 0.819 | 1 | 0.780 | 0.866 | 0.453 | 0.217 | 0.674 | 0.504 |
| **RDM** | 0.479 | 0.756 | -0.365 | -0.445 | -0.246 | -0.031 | 0.458 | -0.206 | -0.605 | -0.734 | 0.079 | 0.561 | 0.795 | 0.780 | 1 | 0.774 | 0.345 | 0.364 | 0.666 | 0.664 |
| **GRM** | 0.253 | 0.507 | -0.645 | 0.039 | -0.292 | -0.387 | -0.164 | -0.637 | -0.948 | -0.418 | -0.364 | 0.219 | 0.518 | 0.866 | 0.774 | 1 | -0.001 | -0.202 | 0.316 | 0.810 |
| **ASL** | 0.922 | 0.851 | -0.410 | -0.254 | 0.784 | 0.914 | 0.238 | -0.052 | -0.092 | -0.691 | 0.405 | 0.925 | 0.827 | 0.453 | 0.345 | -0.001 | 1 | 0.866 | 0.929 | -0.179 |
| **DSM** | 0.665 | 0.691 | 0.064 | -0.652 | 0.503 | 0.816 | 0.661 | 0.420 | 0.239 | -0.743 | 0.736 | 0.760 | 0.710 | 0.217 | 0.364 | -0.202 | 0.866 | 1 | 0.817 | -0.342 |
| **GSM** | 0.930 | 0.976 | -0.493 | -0.350 | 0.533 | 0.712 | 0.347 | -0.150 | -0.328 | -0.827 | 0.327 | 0.960 | 0.970 | 0.674 | 0.666 | 0.316 | 0.929 | 0.817 | 1 | 0.135 |
| **PG** | 0.178 | 0.277 | -0.554 | 0.275 | -0.459 | -0.448 | -0.075 | -0.669 | -0.699 | -0.016 | -0.674 | 0.183 | 0.274 | 0.504 | 0.664 | 0.810 | -0.179 | -0.342 | 0.135 | 1 |

**Table S12.** Multi-scale analysis for landscapes variables at three spatial scales, 2km, 4km and 6km, using linear models for neutral and adaptive quantitative differentiation of *Caryocar brasiliense* among pairs of sampling site of five landscapes in the Cerrado biome, Brazil. HABITAT_2km, habitat (savanna) amount (%) at 2km spatial scale; HABITAT_4km, habitat (savanna) amount (%) at 4km spatial scale; HABITAT_6km, habitat (savanna) amount (%) at 6km spatial scale; SHDI_2KM, compositional heterogeneity at 2km spatial scale; SHDI_4KM, compositional heterogeneity at 4km spatial scale; SHDI_6KM, compositional heterogeneity at 6km spatial scale; LQ_PCA_2km, landscape quality using the PCA resistance value of *C. brasiliense* pollinator and dispersers at 2km spatial scale; LQ_PCA_4km, landscape quality using the PCA resistance value of *C. brasiliense* pollinator and dispersers at 4km spatial scale; LQ_PCA_6km, landscape quality using the PCA resistance value of *C. brasiliense* pollinator and dispersers at 6km spatial scale; *Q_ST_* and *P_ST_*, adaptive quantitative differentiation; STD, seed transversal diameter (mm); SM, seed mass (g); TG, time to germination (days); LL, leaf length (mm); RDM, root dry mass (g); ADM, aboveground dry mass (g); *F_ST__A, G_ST__A* and *Jost_A*, neutral genetic differentiation estimated for adults; *F_IS_*___A, inbreeding coefficient estimated for adults; *F_ST_*_J, *G_ST_*_J and Jost_J, neutral genetic differentiation estimated for juveniles; *F_IS_*_J, inbreeding coefficient estimated for juveniles. The best model for each response variable is in bold.

|  | **multief** | **R2** | **Estimates** | **AIC** |
| --- | --- | --- | --- | --- |
| ***P_ST_*_STD** | **HABITAT_2KM** | **0.028** | **0.000** | **-19.563** |
|  | HABITAT_4KM | 0.014 | 0.000 | -19.492 |
|  | HABITAT_6KM | 0.016 | 0.000 | -19.498 |
| ***P_ST_*_SM** | HABITAT_2KM | 0.125 | -0.001 | -10.317 |
|  | **HABITAT_4KM** | **0.143** | **-0.001** | **-10.423** |
|  | HABITAT_6KM | 0.137 | -0.001 | -10.387 |
| ***Q_ST_*_TG** | HABITAT_2KM | 0.165 | 0.000 | -33.056 |
|  | **HABITAT_4KM** | **0.286** | **0.000** | **-33.837** |
|  | HABITAT_6KM | 0.282 | 0.000 | -33.811 |
| ***Q_ST_*_LL** | **HABITAT_2KM** | **0.013** | **0.000** | **-6.151** |
|  | HABITAT_4KM | 0.001 | 0.000 | -6.087 |
|  | HABITAT_6KM | 0.000 | 0.000 | -6.086 |
| ***Q_ST_*_RDM** | HABITAT_2KM | 0.024 | 0.000 | -6.151 |
|  | HABITAT_4KM | 0.051 | -0.001 | -6.087 |
|  | **HABITAT_6KM** | **0.058** | **-0.001** | **-6.086** |
| ***Q_ST_*_ADM** | HABITAT_2KM | 0.713 | 0.001 | -22.953 |
|  | HABITAT_4KM | 0.731 | 0.001 | -23.284 |
|  | **HABITAT_6KM** | **0.738** | **0.001** | **-23.409** |
| ***F_ST_*_A** | HABITAT_2KM | 0.428 | 0.000 | -33.572 |
|  | HABITAT_4KM | 0.553 | 0.000 | -34.796 |
|  | **HABITAT_6KM** | **0.561** | **0.000** | **-34.890** |
| ***G_ST_*_A** | HABITAT_2KM | 0.210 | 0.000 | -17.570 |
|  | HABITAT_4KM | 0.325 | 0.000 | -18.356 |
|  | **HABITAT_6KM** | **0.332** | **0.000** | **-18.413** |
| **Jost_A** | HABITAT_2KM | 0.003 | 0.000 | -16.642 |
|  | HABITAT_4KM | 0.009 | 0.000 | -16.676 |
|  | **HABITAT_6KM** | **0.010** | **0.000** | **-16.679** |
| ***F_IS_*_A** | **HABITAT_2KM** | **0.007** | **0.000** | **-16.767** |
|  | HABITAT_4KM | 0.000 | 0.000 | -16.732 |
|  | HABITAT_6KM | 0.000 | 0.000 | -16.731 |
| ***F_ST_*_J** | **HABITAT_2KM** | **0.012** | **0.000** | **-31.982** |
|  | HABITAT_4KM | 0.003 | 0.000 | -31.935 |
|  | HABITAT_6KM | 0.004 | 0.000 | -31.945 |
| ***G_ST_*_J** | HABITAT_2KM | 0.087 | 0.000 | -12.920 |
|  | **HABITAT_4KM** | **0.142** | **0.000** | **-13.231** |
|  | HABITAT_6KM | 0.133 | 0.000 | -13.179 |
| **Jost_J** | HABITAT_2KM | 0.114 | 0.000 | -14.349 |
|  | **HABITAT_4KM** | **0.176** | **0.000** | **-14.710** |
|  | HABITAT_6KM | 0.167 | 0.000 | -14.653 |
| ***F_IS_*_J** | **HABITAT_2KM** | **0.007** | **0.000** | **-19.484** |
|  | HABITAT_4KM | 0.004 | 0.000 | -19.470 |
|  | HABITAT_6KM | 0.003 | 0.000 | -19.467 |
|  | **multief** | **R2** | **Estimates** | **AIC** |
| ***P_ST_*_STD** | SHDI_2KM | 0.013 | -0.004 | -19.486 |
|  | SHDI_4KM | 0.022 | -0.005 | -19.532 |
|  | **SHDI_6KM** | **0.043** | **-0.009** | **-19.637** |
| ***P_ST_*_SM** | SHDI_2KM | 0.194 | 0.037 | -10.732 |
|  | **SHDI_4KM** | **0.270** | **0.044** | **-11.223** |
|  | SHDI_6KM | 0.257 | 0.062 | -11.136 |
| ***Q_ST_*_TG** | SHDI_2KM | 0.213 | -0.004 | -33.351 |
|  | SHDI_4KM | 0.304 | -0.005 | -33.970 |
|  | **SHDI_6KM** | **0.326** | **-0.007** | **-34.130** |
| ***Q_ST_*_LL** | SHDI_2KM | 0.001 | 0.004 | -6.090 |
|  | SHDI_4KM | 0.004 | -0.007 | -6.102 |
|  | **SHDI_6KM** | **0.011** | **-0.019** | **-6.141** |
| ***Q_ST_*_RDM** | SHDI_2KM | 0.242 | 0.074 | -5.308 |
|  | SHDI_4KM | 0.155 | 0.060 | -4.769 |
|  | **SHDI_6KM** | **0.243** | **0.106** | **-5.318** |
| ***Q_ST_*_ADM** | **SHDI_2KM** | **0.950** | **-0.041** | **-31.730** |
|  | SHDI_4KM | 0.936 | -0.041 | -30.421 |
|  | SHDI_6KM | 0.923 | -0.058 | -29.537 |
| ***F_ST_*_A** | SHDI_2KM | 0.796 | -0.009 | -38.731 |
|  | SHDI_4KM | 0.796 | -0.009 | -38.735 |
|  | **SHDI_6KM** | **0.880** | **-0.014** | **-41.369** |
| ***G_ST_*_A** | SHDI_2KM | 0.560 | -0.032 | -20.494 |
|  | SHDI_4KM | 0.549 | -0.032 | -20.371 |
|  | **SHDI_6KM** | **0.660** | **-0.050** | **-21.790** |
| **Jost_A** | SHDI_2KM | 0.069 | -0.011 | -16.985 |
|  | SHDI_4KM | 0.082 | -0.012 | -17.057 |
|  | **SHDI_6KM** | **0.149** | **-0.023** | **-17.437** |
| ***F_IS_*_A** | **SHDI_2KM** | **0.023** | **0.006** | **-16.848** |
|  | SHDI_4KM | 0.007 | 0.004 | -16.766 |
|  | SHDI_6KM | 0.004 | 0.004 | -16.752 |
| ***F_ST_*_J** | **SHDI_2KM** | **0.127** | **-0.003** | **-32.599** |
|  | SHDI_4KM | 0.037 | -0.002 | -32.110 |
|  | SHDI_6KM | 0.060 | -0.003 | -32.233 |
| ***G_ST_*_J** | SHDI_2KM | 0.002 | 0.003 | -12.478 |
|  | **SHDI_4KM** | **0.040** | **0.013** | **-12.670** |
|  | SHDI_6KM | 0.021 | 0.013 | -12.572 |
| **Jost_J** | SHDI_2KM | 0.008 | 0.005 | -13.784 |
|  | **SHDI_4KM** | **0.057** | **0.014** | **-14.035** |
|  | SHDI_6KM | 0.033 | 0.015 | -13.911 |
| ***F_IS_*_J** | SHDI_2KM | 0.020 | -0.004 | -19.548 |
|  | SHDI_4KM | 0.049 | -0.007 | -19.702 |
|  | **SHDI_6KM** | **0.086** | **-0.013** | **-19.897** |
|  | **multief** | **R2** | **Estimates** | **AIC** |
| ***P_ST_*_STD** | **LQ_PCA_2KM** | **0.055** | **-0.089** | **-19.704** |
|  | LQ_PCA_4KM | 0.004 | -0.021 | -19.441 |
|  | LQ_PCA_6KM | 0.018 | -0.053 | -19.511 |
| ***P_ST_*_SM** | LQ_PCA_2KM | 0.085 | -0.292 | -10.096 |
|  | **LQ_PCA_4KM** | **0.177** | **-0.353** | **-10.623** |
|  | LQ_PCA_6KM | 0.116 | -0.358 | -10.269 |
| ***Q_ST_*_TG** | LQ_PCA_2KM | 0.202 | 0.047 | -33.283 |
|  | LQ_PCA_4KM | 0.377 | 0.054 | -34.524 |
|  | **LQ_PCA_6KM** | **0.399** | **0.070** | **-34.702** |
| ***Q_ST_*_LL** | LQ_PCA_2KM | 0.004 | -0.090 | -6.104 |
|  | LQ_PCA_4KM | 0.016 | 0.152 | -6.164 |
|  | **LQ_PCA_6KM** | **0.020** | **0.212** | **-6.185** |
| ***Q_ST_*_RDM** | LQ_PCA_2KM | 0.029 | -0.302 | -4.072 |
|  | LQ_PCA_4KM | 0.050 | -0.334 | -4.183 |
|  | **LQ_PCA_6KM** | **0.087** | **-0.547** | **-4.378** |
| ***Q_ST_*_ADM** | LQ_PCA_2KM | 0.640 | 0.395 | -21.823 |
|  | **LQ_PCA_4KM** | **0.712** | **0.350** | **-22.941** |
|  | LQ_PCA_6KM | 0.681 | 0.427 | -22.418 |
| ***F_ST_*_A** | LQ_PCA_2KM | 0.422 | 0.079 | -33.514 |
|  | LQ_PCA_4KM | 0.599 | 0.079 | -35.340 |
|  | **LQ_PCA_6KM** | **0.626** | **0.100** | **-35.690** |
| ***G_ST_*_A** | LQ_PCA_2KM | 0.217 | 0.237 | -17.619 |
|  | LQ_PCA_4KM | 0.375 | 0.262 | -18.745 |
|  | **LQ_PCA_6KM** | **0.415** | **0.345** | **-19.076** |
| **Jost_A** | LQ_PCA_2KM | 0.001 | -0.013 | -16.632 |
|  | LQ_PCA_4KM | 0.032 | 0.075 | -16.794 |
|  | **LQ_PCA_6KM** | **0.043** | **0.109** | **-16.851** |
| ***F_IS_*_A** | LQ_PCA_2KM | 0.000 | 0.000 | -16.730 |
|  | LQ_PCA_4KM | 0.007 | 0.035 | -16.767 |
|  | **LQ_PCA_6KM** | **0.019** | **0.072** | **-16.828** |
| ***F_ST_*_J** | **LQ_PCA_2KM** | **0.005** | **0.007** | **-31.946** |
|  | LQ_PCA_4KM | 0.001 | -0.003 | -31.928 |
|  | LQ_PCA_6KM | 0.000 | 0.000 | -31.922 |
| ***G_ST_*_J** | LQ_PCA_2KM | 0.128 | -0.270 | -13.150 |
|  | **LQ_PCA_4KM** | **0.204** | **-0.286** | **-13.609** |
|  | LQ_PCA_6KM | 0.203 | -0.356 | -13.599 |
| **Jost_J** | LQ_PCA_2KM | 0.162 | -0.267 | -14.627 |
|  | LQ_PCA_4KM | 0.242 | -0.274 | -15.125 |
|  | **LQ_PCA_6KM** | **0.244** | **-0.344** | **-15.140** |
| ***F_IS_*_J** | LQ_PCA_2KM | 0.004 | -0.024 | -19.471 |
|  | **LQ_PCA_4KM** | **0.028** | **0.052** | **-19.590** |
|  | LQ_PCA_6KM | 0.026 | 0.063 | -19.579 |

**Table S13**. Variance Inflation Factor (VIF) at link and node level, for the five landscapes and 10 sites of *Caryocar brasiliense* in the Cerrado biome, Brazil. At link level, the scale of explanatory variables was selected by multi-scale analysis. HABITAT, habitat amount (%); SHDI, compositional heterogeneity; LQ_PCA, landscape quality using the PCA resistance value of *C. brasiliense* pollinator and dispersers; *Q_ST_* and *P_ST_*, adaptive quantitative differentiation; *CV_a_*%, coefficient of additive genetic variation; STD, seed transversal diameter (mm); SM, seed mass (g); TG, time to germination (days); LL, leaf length (mm); RDM, root dry mass (g); ADM, aboveground dry mass (g); *F_ST__*A*, G_ST__*A and *Jost_*A, neutral differentiation estimated for adults; *F_IS_*___A, inbreeding coefficient estimated for adults; *F_ST_*_J, *G_ST_*_J and *Jost*_J, neutral genetic differentiation estimated for juveniles; *F_IS_*_J, inbreeding coefficient estimated for juveniles; *AR*_A, allelic richness estimated for adults; *He_*A, expected heterozygosity estimated for adults; *f*_A, inbreeding coefficient estimated for adults. AR_J, allelic richness estimated for juveniles; *He_*J, expected heterozygosity estimated for juveniles; *f*_J, inbreeding coefficient estimated for juveniles. The selected explanatory variables (VIF<10) for each response variable are in bold.

| **Level** | **Response** | **Est.** | **S.E.** | **t** | **val.** | **p** | **VIF** |
| --- | --- | --- | --- | --- | --- | --- | --- |
| ***Link*** | *P_ST_*_STD | (Intercept) | 1.700 | 1.790 | 0.950 | 0.520 |  |
|  |  | HABITAT_2 | 0.000 | 0.000 | 0.720 | 0.600 | 145.720 |
|  |  | **SHDI_6** | **-0.030** | **0.050** | **-0.620** | **0.650** | **3.690** |
|  |  | LQ_PCA_2 | -2.190 | 2.490 | -0.880 | 0.540 | 129.370 |
|  | *P_ST_*_SM | (Intercept) | 1.770 | 4.370 | 0.400 | 0.760 |  |
|  |  | HABITAT_4 | 0.010 | 0.010 | 0.520 | 0.700 | 84.910 |
|  |  | **SHDI_4** | **0.090** | **0.180** | **0.510** | **0.700** | **7.830** |
|  |  | LQ_PCA_4 | -2.480 | 5.880 | -0.420 | 0.750 | 89.080 |
|  | *Q_ST_*_TG | (Intercept) | -0.490 | 0.190 | -2.630 | 0.230 |  |
|  |  | HABITAT_4 | 0.000 | 0.000 | -2.530 | 0.240 | 60.940 |
|  |  | **SHDI_6** | **0.000** | **0.010** | **-0.200** | **0.870** | **4.330** |
|  |  | LQ_PCA_6 | 0.670 | 0.250 | 2.690 | 0.230 | 63.390 |
|  | *Q_ST_*_LL | (Intercept) | -4.250 | 1.320 | -3.210 | 0.190 |  |
|  |  | HABITAT_2 | -0.010 | 0.000 | -3.660 | 0.170 | 14.220 |
|  |  | **SHDI_6** | **0.070** | **0.100** | **0.710** | **0.610** | **4.440** |
|  |  | LQ_PCA_6 | 5.920 | 1.730 | 3.420 | 0.180 | 19.700 |
|  | *Q_ST_*_RDM | (Intercept) | 3.480 | 7.470 | 0.470 | 0.720 |  |
|  |  | HABITAT_6 | 0.010 | 0.010 | 0.640 | 0.640 | 60.890 |
|  |  | **SHDI_6** | **0.240** | **0.310** | **0.770** | **0.580** | **4.370** |
|  |  | LQ_PCA_6 | -5.140 | 10.060 | -0.510 | 0.700 | 61.330 |
|  | *Q_ST_*_ADM | (Intercept) | 0.080 | 0.570 | 0.130 | 0.920 |  |
|  |  | HABITAT_6 | 0.000 | 0.000 | -0.020 | 0.990 | 81.870 |
|  |  | **SHDI_2** | **-0.040** | **0.020** | **-2.060** | **0.290** | **5.080** |
|  |  | LQ_PCA_4 | -0.010 | 0.780 | -0.010 | 0.990 | 72.410 |
|  | *F_ST_*_A | (Intercept) | -0.190 | 0.060 | -2.960 | 0.210 |  |
|  |  | HABITAT_6 | 0.000 | 0.000 | -3.880 | 0.160 | 60.890 |
|  |  | **SHDI_6** | **-0.020** | **0.000** | **-6.350** | **0.100** | **4.370** |
|  |  | LQ_PCA_6 | 0.300 | 0.080 | 3.540 | 0.180 | 61.330 |
|  | *G_ST_*_A | (Intercept) | -1.330 | 0.510 | -2.630 | 0.230 |  |
|  |  | HABITAT_6 | 0.000 | 0.000 | -3.330 | 0.190 | 60.890 |
|  |  | **SHDI_6** | **-0.070** | **0.020** | **-3.490** | **0.180** | **4.370** |
|  |  | LQ_PCA_6 | 2.030 | 0.680 | 2.980 | 0.210 | 61.330 |
|  | Jost_A | (Intercept) | -2.180 | 0.140 | -15.150 | 0.040 |  |
|  |  | HABITAT_6 | -0.010 | 0.000 | -18.530 | 0.030 | 60.890 |
|  |  | **SHDI_6** | **-0.060** | **0.010** | **-10.780** | **0.060** | **4.370** |
|  |  | LQ_PCA_6 | 3.180 | 0.190 | 16.350 | 0.040 | 61.330 |
|  | *F_IS_*_A | (Intercept) | -1.420 | 0.410 | -3.480 | 0.180 |  |
|  |  | HABITAT_2 | 0.000 | 0.000 | -2.840 | 0.220 | 14.060 |
|  |  | **SHDI_2** | **0.040** | **0.020** | **1.820** | **0.320** | **4.460** |
|  |  | LQ_PCA_6 | 1.960 | 0.550 | 3.540 | 0.180 | 15.950 |
|  | *F_ST_*_J | (Intercept) | 0.210 | 0.770 | 0.270 | 0.830 |  |
|  |  | HABITAT_2 | 0.000 | 0.000 | 0.150 | 0.900 | 169.910 |
|  |  | **SHDI_2** | **-0.010** | **0.020** | **-0.430** | **0.740** | **5.090** |
|  |  | LQ_PCA_2 | -0.240 | 1.070 | -0.220 | 0.860 | 143.800 |
|  | *G_ST_*_J | (Intercept) | 3.900 | 0.350 | 11.120 | 0.060 |  |
|  |  | HABITAT_4 | 0.010 | 0.000 | 8.050 | 0.080 | 84.910 |
|  |  | **SHDI_4** | **-0.110** | **0.010** | **-7.410** | **0.090** | **7.830** |
|  |  | LQ_PCA_4 | -4.940 | 0.470 | -10.470 | 0.060 | 89.080 |
|  | Jost_J | (Intercept) | 2.800 | 1.950 | 1.430 | 0.390 |  |
|  |  | HABITAT_4 | 0.000 | 0.000 | 0.870 | 0.540 | 64.390 |
|  |  | **SHDI_4** | **-0.070** | **0.080** | **-0.970** | **0.510** | **7.500** |
|  |  | LQ_PCA_6 | -3.530 | 2.650 | -1.330 | 0.410 | 61.340 |
|  | *F_IS_*_J | (Intercept) | -0.740 | 0.120 | -5.950 | 0.110 |  |
|  |  | HABITAT_2 | 0.000 | 0.000 | -8.930 | 0.070 | 17.020 |
|  |  | **SHDI_6** | **-0.010** | **0.010** | **-0.810** | **0.570** | **4.660** |
|  |  | LQ_PCA_4 | 1.170 | 0.160 | 7.280 | 0.090 | 24.060 |
| ***Node*** | STD | (Intercept) | 69.660 | 20.470 | 3.400 | 0.020 |  |
|  |  | HABITAT | 0.120 | 0.040 | 2.910 | 0.030 | 313.620 |
|  |  | **SHDI** | **1.830** | **0.340** | **5.410** | **0.000** | **5.410** |
|  |  | ***Ne*** | **0.010** | **0.000** | **2.330** | **0.070** | **1.010** |
|  |  | LQ_PCA | -85.560 | 28.280 | -3.030 | 0.030 | 300.370 |
|  | SM | (Intercept) | 69.660 | 20.470 | 3.400 | 0.020 |  |
|  |  | HABITAT | 0.120 | 0.040 | 2.910 | 0.030 | 313.620 |
|  |  | **SHDI** | **1.830** | **0.340** | **5.410** | **0.000** | **5.410** |
|  |  | ***Ne*** | **0.010** | **0.000** | **2.330** | **0.070** | **1.010** |
|  |  | LQ_PCA | -85.560 | 28.280 | -3.030 | 0.030 | 300.370 |
|  | TG | (Intercept) | 69.660 | 20.470 | 3.400 | 0.020 |  |
|  |  | HABITAT | 0.120 | 0.040 | 2.910 | 0.030 | 313.620 |
|  |  | **SHDI** | **1.830** | **0.340** | **5.410** | **0.000** | **5.410** |
|  |  | ***Ne*** | **0.010** | **0.000** | **2.330** | **0.070** | **1.010** |
|  |  | LQ_PCA | -85.560 | 28.280 | -3.030 | 0.030 | 300.370 |
|  | LL | (Intercept) | 69.660 | 20.470 | 3.400 | 0.020 |  |
|  |  | HABITAT | 0.120 | 0.040 | 2.910 | 0.030 | 313.620 |
|  |  | **SHDI** | **1.830** | **0.340** | **5.410** | **0.000** | **5.410** |
|  |  | ***Ne*** | **0.010** | **0.000** | **2.330** | **0.070** | **1.010** |
|  |  | LQ_PCA | -85.560 | 28.280 | -3.030 | 0.030 | 300.370 |
|  | RDM | (Intercept) | 69.660 | 20.470 | 3.400 | 0.020 |  |
|  |  | HABITAT | 0.120 | 0.040 | 2.910 | 0.030 | 313.620 |
|  |  | **SHDI** | **1.830** | **0.340** | **5.410** | **0.000** | **5.410** |
|  |  | ***Ne*** | **0.010** | **0.000** | **2.330** | **0.070** | **1.010** |
|  |  | LQ_PCA | -85.560 | 28.280 | -3.030 | 0.030 | 300.370 |
|  | ADM | (Intercept) | 69.660 | 20.470 | 3.400 | 0.020 |  |
|  |  | HABITAT | 0.120 | 0.040 | 2.910 | 0.030 | 313.620 |
|  |  | **SHDI** | **1.830** | **0.340** | **5.410** | **0.000** | **5.410** |
|  |  | ***Ne*** | **0.010** | **0.000** | **2.330** | **0.070** | **1.010** |
|  |  | LQ_PCA | -85.560 | 28.280 | -3.030 | 0.030 | 300.370 |
|  | TG_ *CV_a_*% | (Intercept) | 69.660 | 20.470 | 3.400 | 0.020 |  |
|  |  | HABITAT | 0.120 | 0.040 | 2.910 | 0.030 | 313.620 |
|  |  | **SHDI** | **1.830** | **0.340** | **5.410** | **0.000** | **5.410** |
|  |  | ***Ne*** | **0.010** | **0.000** | **2.330** | **0.070** | **1.010** |
|  |  | LQ_PCA | -85.560 | 28.280 | -3.030 | 0.030 | 300.370 |
|  | LL_ *CV_a_*% | (Intercept) | 69.660 | 20.470 | 3.400 | 0.020 |  |
|  |  | HABITAT | 0.120 | 0.040 | 2.910 | 0.030 | 313.620 |
|  |  | **SHDI** | **1.830** | **0.340** | **5.410** | **0.000** | **5.410** |
|  |  | ***Ne*** | **0.010** | **0.000** | **2.330** | **0.070** | **1.010** |
|  |  | LQ_PCA | -85.560 | 28.280 | -3.030 | 0.030 | 300.370 |
|  | RDM_ *CV_a_*% | (Intercept) | 281.480 | 3322.650 | 0.080 | 0.940 |  |
|  |  | HABITAT | 0.690 | 6.580 | 0.100 | 0.920 | 313.620 |
|  |  | **SHDI** | **7.610** | **54.830** | **0.140** | **0.890** | **5.410** |
|  |  | ***Ne*** | **-0.260** | **0.520** | **-0.500** | **0.640** | **1.010** |
|  |  | LQ_PCA | -366.690 | 4591.490 | -0.080 | 0.940 | 300.370 |
|  | ADM_*CV_a_*% | (Intercept) | 2243.560 | 2622.480 | 0.860 | 0.430 |  |
|  |  | HABITAT | 4.540 | 5.190 | 0.870 | 0.420 | 313.620 |
|  |  | **SHDI** | **25.930** | **43.270** | **0.600** | **0.580** | **5.410** |
|  |  | **LQ_PCA** | **0.240** | **0.410** | **0.580** | **0.590** | **1.010** |
|  |  | *Ne* | -3069.940 | 3623.940 | -0.850 | 0.440 | 300.370 |
|  | *AR*_A | (Intercept) | 69.660 | 20.470 | 3.400 | 0.020 |  |
|  |  | HABITAT | 0.120 | 0.040 | 2.910 | 0.030 | 313.620 |
|  |  | **SHDI** | **1.830** | **0.340** | **5.410** | **0.000** | **5.410** |
|  |  | ***Ne*** | **0.010** | **0.000** | **2.330** | **0.070** | **1.010** |
|  |  | LQ_PCA | -85.560 | 28.280 | -3.030 | 0.030 | 300.370 |
|  | *He*_A | (Intercept) | 1.560 | 2.430 | 0.640 | 0.550 |  |
|  |  | HABITAT | 0.000 | 0.000 | 0.260 | 0.810 | 313.620 |
|  |  | **SHDI** | **0.060** | **0.040** | **1.490** | **0.200** | **5.410** |
|  |  | ***Ne*** | **0.000** | **0.000** | **0.920** | **0.400** | **1.010** |
|  |  | LQ_PCA | -1.090 | 3.360 | -0.330 | 0.760 | 300.370 |
|  | *f*_A | (Intercept) | -4.840 | 5.150 | -0.940 | 0.390 |  |
|  |  | HABITAT | -0.010 | 0.010 | -0.930 | 0.400 | 313.620 |
|  |  | **SHDI** | **-0.030** | **0.080** | **-0.340** | **0.750** | **5.410** |
|  |  | ***Ne*** | **0.000** | **0.000** | **-0.040** | **0.970** | **1.010** |
|  |  | LQ_PCA | 6.660 | 7.110 | 0.940 | 0.390 | 300.370 |
|  |  |  |  |  |  |  |  |
|  | AR_J | (Intercept) | 45.180 | 60.230 | 0.750 | 0.490 |  |
|  |  | HABITAT | 0.070 | 0.120 | 0.570 | 0.600 | 313.620 |
|  |  | **SHDI** | **1.540** | **0.990** | **1.550** | **0.180** | **5.410** |
|  |  | ***Ne*** | **-0.010** | **0.010** | **-1.060** | **0.340** | **1.010** |
|  |  | LQ_PCA | -50.420 | 83.230 | -0.610 | 0.570 | 300.370 |
|  | *He*_J | (Intercept) | 4.340 | 3.530 | 1.230 | 0.270 |  |
|  |  | HABITAT | 0.010 | 0.010 | 0.900 | 0.410 | 313.620 |
|  |  | **SHDI** | **0.080** | **0.060** | **1.320** | **0.250** | **5.410** |
|  |  | ***Ne*** | **0.000** | **0.000** | **-1.020** | **0.350** | **1.010** |
|  |  | LQ_PCA | -4.870 | 4.870 | -1.000 | 0.360 | 300.370 |
|  | *f*_J | (Intercept) | 1.870 | 4.010 | 0.470 | 0.660 |  |
|  |  | HABITAT | 0.000 | 0.010 | 0.470 | 0.660 | 313.620 |
|  |  | **SHDI** | **0.040** | **0.070** | **0.600** | **0.580** | **5.410** |
|  |  | ***Ne*** | **0.000** | **0.000** | **-1.180** | **0.290** | **1.010** |
|  |  | LQ_PCA | -2.510 | 5.540 | -0.450 | 0.670 | 300.370 |

**Table S14.** Additive genetic coefficient of variation (*CV_a_*%), narrow-sense heritability (*h^2^*), additive variance (*V_a_*) and residual variance (*Rv*) of adaptive quantitative traits of *Caryocar brasiliense* for each sampling site. The traits were measured in seedlings obtained from controlled experiment in nursery from seeds collected from 10 sites in the Cerrado biome, Brazil. TG, time to germination (days); LL, leaf length (mm); RDM, root dry mass (g); ADM, aboveground dry mass (g).

|  | **TG** | | | | **LL** | | | | **RDM** | | | | **ADM** | | | |
| --- | --- | --- | --- | --- | --- | --- | --- | --- | --- | --- | --- | --- | --- | --- | --- | --- |
| **Site** | ***CV_a_%*** | ***h^2^*** | ***V_a_*** | ***R_V_ (q2)*** | ***CV_a_%*** | ***h^2^*** | ***V_a_*** | ***R_V_ (q2)*** | ***CV_a_%*** | ***h^2^*** | ***V_a_*** | ***R_V_ (q2)*** | ***CV_a_%*** | ***h^2^*** | ***V_a_*** | ***R_V_ (q2)*** |
| **L1F1** | 45.554 | 0.737 | 516.695 | 313.662 | 5.786 | 0.218 | 20.067 | 77.169 | 35.036 | 0.437 | 1.301 | 2.001 | 30.985 | 0.627 | 0.686 | 0.579 |
| **L1F2** | 29.959 | 0.883 | 200.061 | 76.503 | 1.995 | 0.016 | 2.258 | 143.708 | 3.227 | 0.006 | 0.010 | 1.692 | 35.283 | 0.534 | 0.809 | 0.910 |
| **L2F3** | 39.099 | 0.902 | 223.951 | 80.279 | 12.047 | 0.867 | 82.579 | 33.281 | 4.101 | 0.011 | 0.024 | 2.080 | 15.290 | 0.275 | 0.163 | 0.470 |
| **L2F4** | 37.518 | 0.935 | 286.013 | 91.235 | 11.359 | 0.733 | 69.731 | 42.794 | 1.885 | 0.003 | 0.005 | 1.512 | 11.053 | 0.060 | 0.116 | 1.844 |
| **L3F5** | 48.393 | 1.000 | 430.041 | 54.901 | 6.819 | 0.533 | 30.131 | 33.892 | 3.460 | 0.016 | 0.016 | 0.951 | 13.543 | 0.303 | 0.170 | 0.434 |
| **L3F6** | 4.616 | 0.081 | 3.269 | 37.767 | 2.538 | 0.081 | 4.276 | 49.409 | 8.032 | 0.081 | 0.054 | 0.625 | 9.861 | 0.081 | 0.069 | 0.799 |
| **L4F7** | 3.330 | 0.003 | 2.710 | 915.067 | 1.367 | 0.008 | 1.001 | 117.611 | 60.406 | 0.841 | 2.602 | 1.144 | 53.961 | 0.858 | 1.468 | 0.610 |
| **L4F8** | 39.432 | 0.405 | 369.175 | 634.682 | 6.462 | 0.212 | 25.150 | 99.899 | 22.993 | 0.299 | 0.606 | 1.575 | 1.633 | 0.003 | 0.002 | 0.622 |
| **L5F9** | 46.720 | 1.000 | 544.021 | 119.788 | 13.143 | 0.466 | 101.767 | 142.050 | 20.352 | 0.324 | 0.483 | 1.129 | 27.020 | 0.493 | 0.686 | 0.878 |
| **L5F10** | 1.288 | 0.004 | 0.307 | 71.514 | 18.841 | 0.613 | 161.860 | 142.540 | 46.242 | 0.593 | 2.309 | 2.161 | 50.180 | 0.569 | 1.326 | 1.334 |

**Table S15.** Genetic differentiation of adaptive quantitative traits of *Caryocar brasiliense* measured in seeds (*P_ST_*) and seedlings (*Q_ST_*) from 10 sites in the Cerrado biome, Brazil. STD, seed transversal diameter (mm); SM, seed mass (g); TG, time to germination (days); LL, leaf length (mm); RDM, root dry mass (g); ADM = aboveground dry mass (g).

|  | *P_ST_* | | *Q_ST_* | | | |
| --- | --- | --- | --- | --- | --- | --- |
| **Landscape** | **STD** | **SM** | **TG** | **LL** | **DRM** | **ADM** |
| **L1** | 0.001 | 0.170 | 0.001 | 0.002 | 0.001 | 0.001 |
| **L2** | 0.058 | 0.045 | 0.001 | 0.001 | 0.000 | 0.036 |
| **L3** | 0.042 | 0.039 | 0.000 | 0.002 | 0.230 | 0.010 |
| **L4** | 0.043 | 0.075 | 0.012 | 0.188 | 0.027 | 0.004 |
| **L5** | 0.031 | 0.035 | 0.011 | 0.058 | 0.001 | 0.067 |

**Table S16.** Neutral genetic estimates for adults and juveniles sampled in 10 sites of *Caryocar brasiliense* in Cerrado biome. Brazil. *Ne,* effective population size; *AR*, allelic richness; *He*, expected heterozygosity; *f*, inbreeding coefficient.

| **Site** | **Life Stage** | ***AR*** | ***He*** | ***Ne*** | ***f (p)*** |
| --- | --- | --- | --- | --- | --- |
| **L1F1** | **Adults** | 9.087 | 0.818 | 24.800 | 0.088 (0.000) |
| **L1F2** |  | 9.332 | 0.845 | 65.000 | 0.004 (0.808) |
| **L2F3** |  | 7.974 | 0.790 | 15.600 | -0.004 (0.831) |
| **L2F4** |  | 9.012 | 0.825 | 11.100 | 0.004 (0.791) |
| **L3F5** |  | 8.941 | 0.836 | 12.200 | 0.029 (0.081) |
| **L3F6** |  | 9.127 | 0.851 | 13.600 | 0.001 (0.947) |
| **L4F7** |  | 8.991 | 0.806 | 12.600 | 0.051 (0.002) |
| **L4F8** |  | 9.022 | 0.840 | 19.200 | 0.090 (0.000) |
| **L5F9** |  | 8.565 | 0.807 | 39.100 | 0.054 (0.013) |
| **L5F10** |  | 8.099 | 0.767 | 13.800 | 0.003 (0.883) |
| **L1F1** | **Juveniles** | 10.192 | 0.816 | 38.600 | 0.052 (0.005) |
| **L1F2** |  | 9.265 | 0.798 | 14.100 | 0.020 (0.295) |
| **L2F3** |  | 9.042 | 0.814 | 15.900 | 0.024 (0.232) |
| **L2F4** |  | 9.988 | 0.846 | 14.300 | 0.096 (0.000) |
| **L3F5** |  | 10.235 | 0.828 | 27.500 | 0.048 (0.014) |
| **L3F6** |  | 9.874 | 0.851 | 35.000 | 0.054 (0.029) |
| **L4F7** |  | 9.156 | 0.824 | 8.300 | 0.082 (0.006) |
| **L4F8** |  | 9.514 | 0.809 | 16.600 | 0.105 (0.000) |
| **L5F9** |  | 8.769 | 0.792 | 15.200 | 0.067 (0.017) |
| **L5F10** |  | 8.676 | 0.740 | 18.300 | 0.062 (0.062) |

**Table S17**. Neutral genetic differentiation and inbreeding coefficient for adults and juveniles sampled in pairs of sampling sites within five landscapes of *Caryocar brasiliense* in Cerrado biome, Brazil.

| **Landscape** | **Life Stage** | ***F_ST_ (p)*** | ***G_ST_'* *(p)*** | **Jost'*D* (*p*)** | ***F_IS_ (p)*** |
| --- | --- | --- | --- | --- | --- |
| **L1** | **Adults** | 0.008 (0.001) | 0.049 (0.001) | 0.037 (0.004) | 0.048 (0.000) |
| **L2** |  | 0.014 (0.001) | 0.070 (0.001) | 0.058 (0.002) | 0.007 (0.001) |
| **L3** |  | 0.005 (0.041) | 0.021 (0.041) | 0.018 (0.007) | 0.017 (0.003) |
| **L4** |  | 0.014 (0.001) | 0.080 (0.001) | 0.093 (0.001) | 0.078 (0.000) |
| **L5** |  | 0.022 (0.001) | 0.095 (0.001) | 0.061 (0.007) | 0.040 (0.000) |
| **L1** | **Juveniles** | 0.026 (0.009) | 0.130 (0.001) | 0.108 (0.002) | 0.047 (0.000) |
| **L2** |  | 0.028 (0.001) | 0.170 (0.001) | 0.146 (0.001) | 0.070 (0.000) |
| **L3** |  | 0.016 (0.001) | 0.103 (0.001) | 0.089 (0.002) | 0.057 (0.000) |
| **L4** |  | 0.014 (0.022) | 0.061 (0.012) | 0.051 (0.036) | 0.103 (0.000) |
| **L5** |  | 0.022 (0.002) | 0.081 (0.002) | 0.064 (0.001) | 0.074 (0.000) |

**Table S18.** Model selection using linear models to describe the relation of explanatory variables at node level and the patterns additive quantitative and neutral genetic parameters in ten sampling sites of *Caryocar brasiliense*, in Cerrado biome, Brazil. The models with ΔAIC < 2 are in bold. STD, seed transversal diameter (mm); SM, seed mass (g); TG, time to germination (days); LL, leaf length (mm); RDM, root dry mass (g); ADM = aboveground dry mass (g); *AR*, allelic richness; *He*, expected heterozygosity; *f*, inbreeding coefficient.

| **Model** | **Mean - STD** | | | | |
| --- | --- | --- | --- | --- | --- |
|  | **AICc** | **ΔAICc** | **wAIC** | **p** | ***β*** |
| **Null model** | **20.600** | **0.000** | **0.790** | **-** | **-** |
| Compositional heterogeneity (SHDI) | 24.500 | 3.900 | 0.114 | 0.578 | -0.288 |
| Effective population size *(Ne)* | 24.800 | 4.200 | 0.096 | 0.824 | 0.003 |
|  | **Mean - SM** | | | | |
|  | **AICc** | **ΔAICc** | **wAIC** | **p** | ***β*** |
| **Null model** | **32.100** | **0.000** | **0.805** | **-** | **-** |
| Compositional heterogeneity (SHDI) | 36.300 | 4.200 | 0.099 | 0.783 | 0.256 |
| Effective population size *(Ne)* | 36.400 | 4.300 | 0.096 | 0.893 | 0.003 |
|  | **Mean - TG** | | | | |
|  | **AICc** | **ΔAICc** | **wAIC** | **p** | ***β*** |
| **Null model** | **61.100** | **0.000** | **0.790** | **-** | **-** |
| Compositional heterogeneity (SHDI) | 64.900 | 3.900 | 0.114 | 0.577 | 2.186 |
| Effective population size *(Ne)* | 65.300 | 4.200 | 0.096 | 0.808 | 0.021 |
|  | **Mean - LL** | | | | |
|  | **AICc** | **ΔAICc** | **wAIC** | **p** | ***β*** |
| **Null model** | **62.500** | **0.000** | **0.525** | **-** | **-** |
| **Compositional heterogeneity (SHDI)** | **63.000** | **0.500** | **0.410** | **0.091** | **6.539** |
| Effective population size *(Ne)* | 66.600 | 4.200 | 0.065 | 0.762 | 28.000 |
|  | **Mean - RDM** | | | | |
|  | **AICc** | **ΔAICc** | **wAIC** | **p** | ***β*** |
| **Null model** | **9.000** | **0.000** | **0.580** | **-** | **-** |
| **Compositional heterogeneity (SHDI)** | **10.200** | **1.200** | **0.310** | **0.130** | **-0.412** |
| Effective population size *(Ne)* | 12.400 | 3.400 | 0.110 | 0.408 | -0.005 |
|  | **Mean - ADM** | | | | |
|  | **.** | **ΔAICc** | **wAIC** | **p** | ***β*** |
| **Null model** | **9.400** | **0.000** | **0.794** | **-** | **-** |
| Effective population size *(Ne)* | 13.300 | 3.900 | 0.112 | 0.910 | -0.033 |
| Compositional heterogeneity (SHDI) | 13.700 | 4.300 | 0.094 | 0.594 | -0.003 |
|  | **CVa% - TG** | | | | |
|  | **AICc** | **ΔAICc** | **wAIC** | **p** | ***β*** |
| **Null model** | **92.000** | **0.000** | **0.786** | **-** | **-** |
| Effective population size *(Ne)* | 95.700 | 3.700 | 0.122 | 0.980 | -0.468 |
| Compositional heterogeneity (SHDI) | 96.300 | 4.300 | 0.092 | 0.517 | 0.259 |
|  | **CVa% - LL** | | | | |
|  | **AICc** | **ΔAICc** | **wAIC** | **p** | ***β*** |
| **Compositional heterogeneity (SHDI)** | **64.800** | **0.000** | **0.795** | **0.019** | **-10.915** |
| Null model | 67.800 | 3.000 | 0.177 | - | - |
| Effective population size *(Ne)* | 71.500 | 6.700 | 0.028 | 0.517 | -0.077 |
|  | **CVa% - RDM** | | | | |
|  | **AICc** | **ΔAICc** | **wAIC** | **p** | ***β*** |
| **Null model** | **93.600** | **0.000** | **0.787** | **-** | **-** |
| Effective population size *(Ne)* | 97.300 | 3.800 | 0.120 | 0.970 | 0.750 |
| Compositional heterogeneity (SHDI) | 97.900 | 4.300 | 0.092 | 0.530 | -0.273 |
|  | **CVa% - ADM** | | | | |
|  | **AICc** | **ΔAICc** | **wAIC** | **p** | ***β*** |
| **Null model** | **90.500** | **0.000** | **0.780** | **-** | **-** |
| Effective population size *(Ne)* | 94.400 | 3.900 | 0.110 | 0.607 | -0.879 |
| Compositional heterogeneity (SHDI) | 94.400 | 3.900 | 0.110 | 0.587 | -0.203 |
|  | **Adults - *AR*** | | | | |
|  | **AICc** | **dAICc** | **wAIC** | **p** | ***β*** |
| **Compositional heterogeneity (SHDI)** | **10.500** | **0.000** | **0.960** | **0.004** | **0.990** |
| Null model | 17.200 | 6.700 | 0.033 | - | - |
| Effective population size *(Ne)* | 20.500 | 10.000 | 0.007 | 0.381 | 0.008 |
|  | **Adults - *He*** | | | | |
|  | **AICc** | **dAICc** | **wAIC** | **p** | ***β*** |
| **Compositional heterogeneity (SHDI)** | **-43.600** | **0.000** | **0.867** | **0.012** | **0.053** |
| Null model | -39.600 | 4.100 | 0.113 | - | - |
| Effective population size *(Ne)* | -36.000 | 7.600 | 0.019 | 0.456 | 0.000 |
|  | **Adults - *f*** | | | | |
|  | **AICc** | **dAICc** | **wAIC** | **p** | ***β*** |
| **Null model** | **-33.100** | **0.000** | **0.699** | **-** | **-** |
| Compositional heterogeneity (SHDI) | -30.800 | 2.300 | 0.219 | 0.223 | 0.041 |
| Effective population size *(Ne)* | -28.900 | 4.300 | 0.082 | 0.980 | 0.000 |
|  | **Juveniles - *AR*** | | | | |
|  | **AICc** | **dAICc** | **wAIC** | **p** | ***β*** |
| **Compositional heterogeneity (SHDI)** | **20.000** | **0.000** | **0.490** | **0.029** | **1.054** |
| **Effective population size *(Ne)*** | **20.800** | **0.800** | **0.330** | **0.041** | **0.038** |
| Null model | 22.000 | 2.000 | 0.180 | - | - |
|  | **Juveniles - *He*** | | | | |
|  | **AICc** | **dAICc** | **wAIC** | **p** | ***β*** |
| **Null model** | **-36.100** | **0.000** | **0.690** | **-** | **-** |
| Compositional heterogeneity (SHDI) | -33.500 | 2.600 | 0.190 | 0.257 | 0.033 |
| Effective population size *(Ne)* | -32.600 | 3.500 | 0.120 | 0.454 | 0.000 |
|  | **Juveniles - *f*** | | | | |
|  | **AICc** | **dAICc** | **wAIC** | **p** | ***β*** |
| **Null model** | **-38.600** | **0.000** | **0.777** | **-** | **-** |
| Effective population size *(Ne)* | -34.900 | 3.700 | 0.123 | 0.722 | 0.010 |
| Compositional heterogeneity (SHDI) | -34.500 | 4.100 | 0.099 | 0.500 | 0.000 |

**Table S19.** Linear models at link level for additive quantitative and neutral genetic differentiation among pairs of 10 sampling site of *Caryocar brasiliense* in five landscapes in the Cerrado biome, Brazil. The models with p < 0.05 are in bold.

| **Genetic variable** | **Explanatory variable** | ***p*** | ***β*** | **AIC** |
| --- | --- | --- | --- | --- |
| *P_ST_* STD | Compositional heterogeneity 6km | 0.739 | -0.009 | -19.637 |
| *P_ST_* SM | Compositional heterogeneity 4km | 0.370 | 0.044 | -11.223 |
| *Q_ST_* TG | Compositional heterogeneity 6km | 0.315 | -0.007 | -34.130 |
| *Q_ST_* LL | Compositional heterogeneity 6km | 0.865 | -0.019 | -6.141 |
| *Q_ST_* RDM | Compositional heterogeneity 6km | 0.399 | 0.107 | -5.318 |
| ***Q_ST_* ADM** | **Compositional heterogeneity 2km** | **0.005** | **-0.04** | **-31.730** |
| ***F_ST_* Adults** | **Compositional heterogeneity 6km** | **0.018** | **-0.014** | **-41.369** |
| *G_ST_* Adults | Compositional heterogeneity 6km | 0.095 | -0.05 | -21.790 |
| *Jost'D* Adults | Compositional heterogeneity 6km | 0.521 | -0.023 | -17.437 |
| *F_IS_* Adults | Compositional heterogeneity 2km | 0.806 | 0.006 | -16.848 |
| *F_ST_* Juveniles | Compositional heterogeneity 2km | 0.557 | -0.03 | -32.599 |
| *G_ST_* Juveniles | Compositional heterogeneity 4km | 0.747 | 0.013 | -12.670 |
| *Jost'D* Juveniles | Compositional heterogeneity 4km | 0.700 | 0.014 | -14.035 |
| *F_IS_* Juveniles | Compositional heterogeneity 6km | 0.633 | -0.013 | -19.897 |
