# Supplementary material for: Agricultural Landscape Heterogeneity Matter: Responses of Neutral Genetic Diversity and Adaptive Traits in a Neotropical Savanna Tree
Source: Front Genet. 2021 Feb 4;11:606222. doi: 10.3389/fgene.2020.606222 (PMC7890196; doi:10.3389/fgene.2020.606222)
Supplement: Supplementary file 2 [file Data_Sheet_2.docx]

**Agricultural landscape heterogeneity matters: responses of neutral genetic diversity and adaptative trait in a Neotropical savanna tree**

Tatiana Souza do Amaral, Juliana Silveira dos Santos, Fernanda Fraga Rosa, Marcelo Bruno Pessôa, Lázaro José Chaves, Milton Cezar Ribeiro, Rosane Garcia Collevatti

**Appendix S2 - Figures**

**(a)**

**
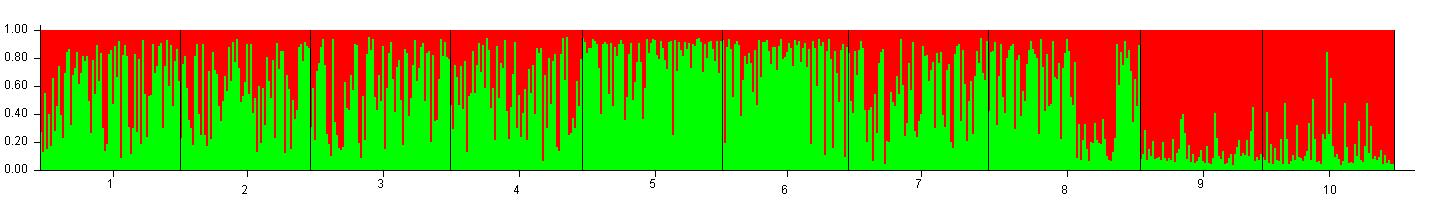
**

**(b)**

**Figure S1.** Bayesian clustering simulation for adults. (a) Rate of change of delta K in relation to K. Delta K = mean (|L”(K)|)/sd(L(K)); (b) coancestry plot for K = 2.

**(a)**

**
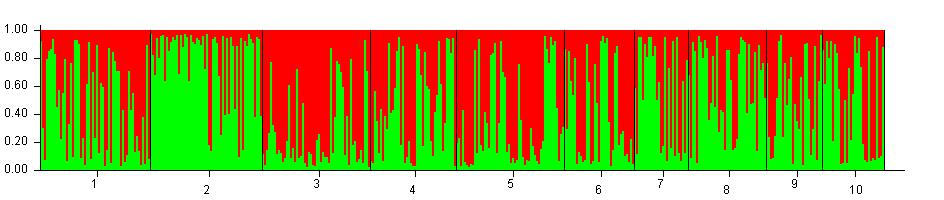
**

**(b)**

**Figure S2.** Bayesian clustering simulation for juveniles. (a) Rate of change of delta K in relation to K. Delta K = mean (|L”(K)|)/sd(L(K)); (b) coancestry plot for K = 2.
